# Triflate Salts as Alternative Non-Chlorinated Oxidants for the Oxidative Chemical Vapor Deposition and Electronic Engineering of Conjugated Polymers

**DOI:** 10.1021/acs.macromol.4c01183

**Published:** 2024-10-02

**Authors:** Drialys Cardenas-Morcoso, Justine Debard, Faezeh Farzin, Nicolas D. Boscher

**Affiliations:** Material Research and Technology Department, Luxembourg Institute of Science and Technology, 28 Avenue des Hauts-Fourneaux, 4362 Esch-sur-Alzette, Luxembourg

## Abstract

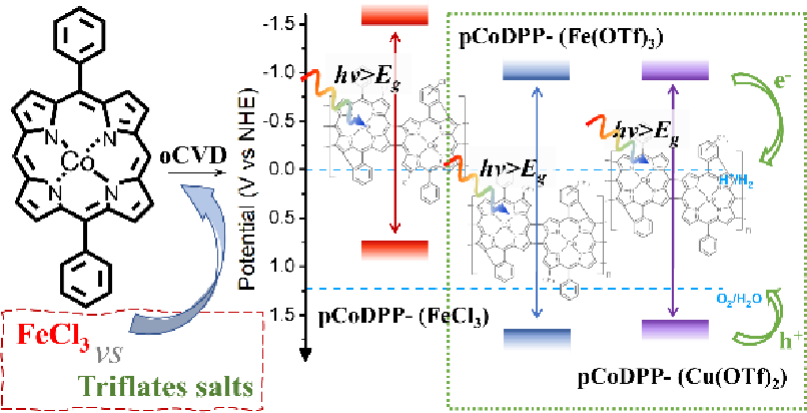

Oxidative chemical vapor deposition (oCVD) stands as an attractive approach for the synthesis, engineering, and integration of conjugated polymers for advanced electronic and optoelectronic applications. In oCVD, the oxidant significantly influences the conformational and optoelectronic properties of the resulting conjugated polymer thin films. In this work, triflate salts of iron(III) and copper(II) (Fe(OTf)_3_ and Cu(OTf)_2_, respectively) are investigated for the first time as suitable alternative oxidants to the widely used iron(III) chloride (FeCl_3_) for the oCVD of conjugated polymers. Structural and compositional characterizations of the resulting thin films evidenced the successful polymerization of cobalt(II) 5,15-diphenyl porphyrin using either Fe(OTf)_3_ or Cu(OTf)_2_. Along with an intermolecular dehydrogenative C–C coupling reaction, the occurrence of side reactions, such as the inclusion of −CF_3_ groups and demetalation and subsequent insertion of copper(II) in the porphyrin macrocycle when using Cu(OTf)_2_, were evidenced. Interestingly, the inclusion of −CF_3_ groups into the polymer backbone when using triflate salts results in a deepening of the frontier energy levels, while the insertion of copper(II) contributes to a reduction in the band gap energy. This work demonstrates that the careful selection of the oxidant agent in oCVD enables fine-tuning the optoelectronic properties of conjugated polymers to suit specific application requirements.

## Introduction

Conjugated polymers represent a class of materials characterized by an extended π-electron system that confers outstanding electronic and optical properties, with extensive applications in optoelectronics and energy devices. The semiconducting nature of conjugated polymers makes them essential in electronic devices like organic solar cells,^1,2^ field-effect transistors,^3^ and light-emitting diodes.^4−6^ Beyond, conjugated polymers with metal-like conductivity represent the next generation of organic electronic devices used in smart technologies.^7,8^ Additionally, owing to their synthetic tunability and adjustable function, conjugated polymers have met an even broader large range of applications, including catalysis and solar-driven chemical transformation^9,10^ and sensing devices.^11−13^

The synthesis of conjugated polymers mainly relies on a variety of solution-based methods, allowing control of the conjugated polymer chain’s structure and properties. For instance, Sonogashira cross-coupling polymerization is a widely employed technique that involves the coupling of terminal alkynes with aryl halides under palladium catalysis.^14,15^ Alternatively, Suzuki coupling implies the cross-coupling of aryl boronic acids with aryl halides, facilitated by palladium catalysis, enabling the synthesis of conjugated polymers with high molecular weight and well-defined structures.^16−18^ On the other hand, electropolymerization consists of simultaneous electrochemical oxidation and polymerization of monomers reacting at the anode of an electrochemical cell.^19,20^ Electropolymerization affords a much wider choice of dopant ions for the conjugated polymers from the selection of appropriate electrochemical conditions such as the electrolyte and oxidation potential. One of the first electron-conducting conjugated polymers based on fused porphine units was prepared by Vorotyntsev et al. using electro-oxidation at very low potential.^21^ In contrast to the aforementioned methods, the oxidative intermolecular dehydrogenative coupling route allows direct polymerization via oxidative coupling reactions, eliminating the need for prefunctionalized monomers.^22^ In this process, an oxidant reagent promotes the formation of radical cations from electron-rich monomers. The radical cations thus couple, after which deprotonation gives a neutral dimer. Subsequently, polymerization proceeds via a step-growth mechanism involving sequential oxidation, coupling, and deprotonation steps.^23^

Notably, conjugated polymers are often weakly soluble or even insoluble, requiring the inclusion of solubilizing groups, the use of soluble molecular dopants, or the use of unsafe and costly solvents for their processing. Such a drawback is overcome through vapor-phase synthesis approaches, such as the oxidative chemical vapor deposition (oCVD).^1,24,25^ In this method, the monomer and oxidant are delivered from the vapor-phase to a substrate, on which they polymerize via oxidative coupling reactions and readily form a conjugated polymer thin film on a substrate surface. The oCVD process offers precise control over film thickness, morphology, and chemical composition, making it suitable for the preparation of high-quality conjugated polymer thin films with uniform coverage over large areas and eliminating the need for solvents, solubilizing groups, or the addition of soluble molecular dopants.^24,26^ For instance, polystyrene-sulfone (PSS) is often added to poly(3,4-ethylenedioxythiophene) (PEDOT) to form the solvable complex PEDOT:PSS. Instead, the oCVD method suppresses the need to add the PSS dopant,^27^ enabling conductivity values as high as 6500 S·cm^–1^.^28^ Notably, the oCVD approach has been successfully used for the preparation of thin films of polythiophenes,^29^ polypyrroles,^30^ and poly(diketopyrrolopyrrole) derivatives,^31^ among other conjugated polymers.^24^ Moreover, the ability to tailor the thin film properties through the control of deposition parameters, such as monomer structure,^32^ substrate temperature,^33,34^ and gas flow rates, further endorses the oCVD approach for the synthesis and engineering of conjugated polymers with tailored properties.

**Scheme 1:**
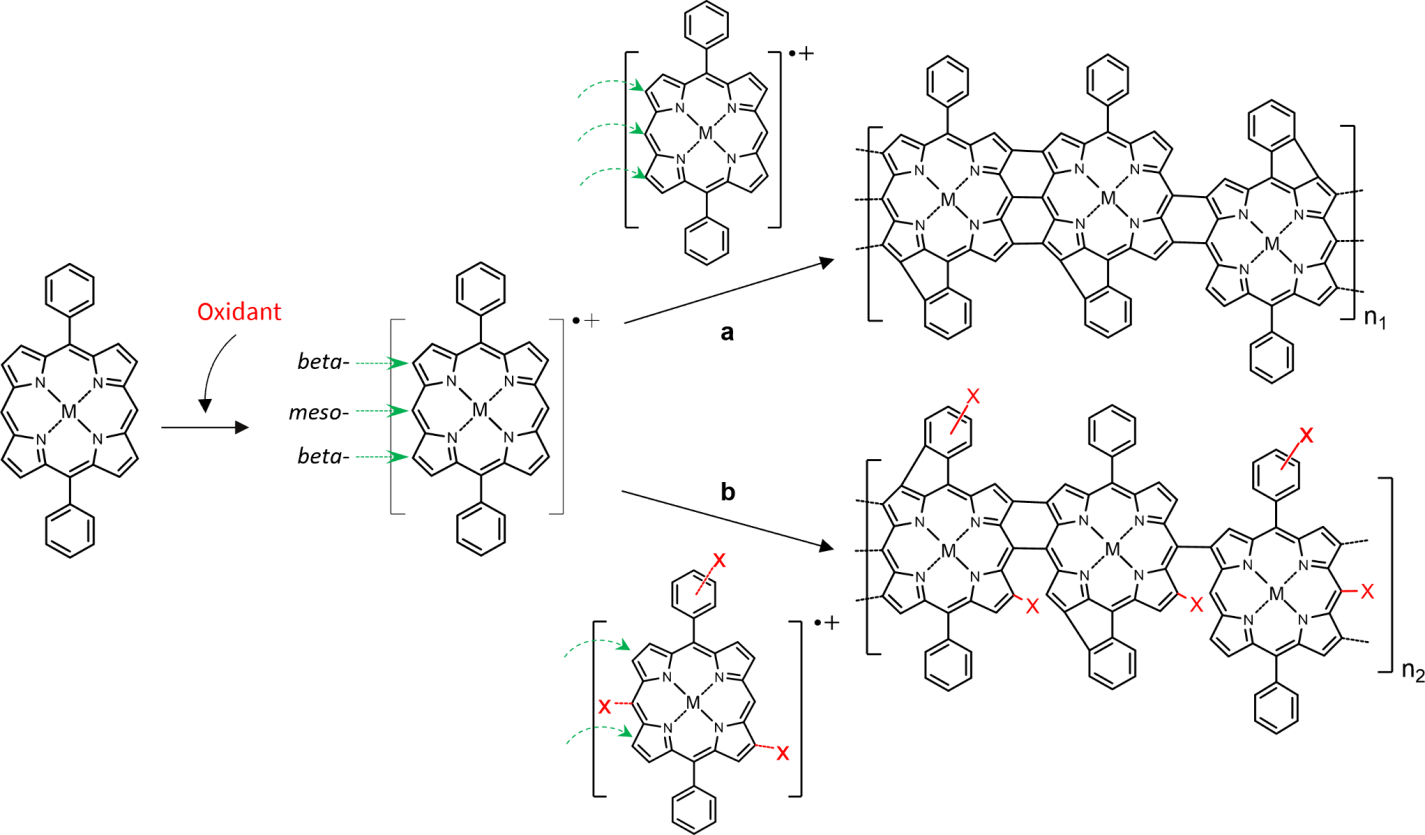
Representation of the Intermolecular and Intramolecular Dehydrogenative C–C Coupling Reaction Taking Place during the oCVD Process and Possible Side Reactions (a) The formation of singly and/or doubly and/or triply fused metalloporphyrin conjugated polymers. (b) Possible side reactions include the integration of the oxidant’s counterion into one of the free *beta-* and *meso-*positions of the porphyrin macrocycle, blocking the intermolecular and intramolecular dehydrogenative C–C coupling reaction from these sites, or into the available position of the aryl substituent.

In this context, oCVD has enabled the simultaneous synthesis and deposition of conjugated polymers from complex polycyclic monomers, such as metalloporphyrins,^35−37^ in view of granting highly advanced electronic and optoelectronic properties attractive for catalysis^32,38,39^ and sensing^40^ applications. Bengasi et al. reported for the first time the formation of fused metalloporphyrin conjugated polymer thin films by oCVD.^35^ In this process, one-electron oxidation of the metalloporphyrin monomer forms a radical cation species with the removal of a proton, followed by the intermolecular dehydrogenative C–C coupling of this radical cation with a neutral metalloporphyrin through the free *beta-* and *meso-*positions of the porphyrin macrocycle, forming a singly *beta-meso* or *meso-meso* linked dimer. Subsequent oxidative couplings lead to the formation of double or triple links between the metalloporphyrin units and the step-growth of fused metalloporphyrin conjugated polymer chains (Scheme 1a). Based on the oCVD reactivity of metalloporphyrins, it is possible to tune the molecular conformation of the resulting conjugated polymers, ultimately engineering their electronic and optoelectronic properties, such as conductivity and energy band level arrangement, with a significant impact on their potential implementation in photo- and electrocatalytic applications.^32,39^

Due to its high oxidation potential (*E*^0^(Fe^3+^/Fe^2+^) = 0.77 V) and suitable volatility, iron(III) chloride (FeCl_3_) have been preferentially used as the oxidant agent in oCVD processing of conjugated polymers,^24^ including fused metalloporphyrin conjugated polymer thin films.^35,37^ Alternatively, copper(II) chloride (CuCl_2_) and copper(II) perchlorate (Cu(ClO_4_)_2_) have been also reported as oxidants in the oCVD reaction to form conjugated polymers.^24,41^ However, the weaker oxidizing strength of copper(II) (*E*^0^(Cu^2+^/Cu^+^) = 0.33 V) leads to a polymerization ratio lower than that when using FeCl_3_.^24,41^ In addition, highly volatile oxidants such as vanadium oxytrichloride (VOCl_3_) and antimony pentachloride (SbCl_5_) have also been successfully investigated,^24,42,43^ in all cases, including the aforementioned solid oxidants, leading to the formation of chlorinated species.^44^ Notably, the chlorination of a conjugated polymer backbone can significantly impact the resulting energy band structure and hence the optoelectronic properties and performances.^45,46^ Moreover, chlorine is known as a poor leaving group for aromatic rings; thus, it is reasonable to assume that chlorination of *meso-* and *beta-*positions of the porphyrin macrocycle will prevent the formation of large fused metalloporphyrin conjugated polymer chains, as represented in the Scheme 1b.

In order to prevent the formation of chlorinated byproducts, Lewtak et al. successfully used iron(III) perchlorate (Fe(ClO_4_)_3_) and iron(III) trifluoromethane sulfonate (aka iron(III) triflate, Fe(OTf)_3_) in the solution-based oxidative C–C coupling synthesis of naphthalene-fused porphyrins, replacing the commonly used FeCl_3_ as the oxidizing reagent.^47^ Moreover, Feng et al. used Fe(OTf)_3_ in the development of a general oxidative C–C coupling method for the synthesis of doubly or triply linked metalloporphyrin dimers.^48^ Interestingly, Fe(OTf)_3_ was previously used for the oxidative chemical polymerization and stabilization of PEDOT via a conventional chemical pathway,^49,50^ as well as in the vapor-phase polymerization (VPP) synthesis of PEDOT:OTf,^51^ making Fe(OTf)_3_ a potential alternative to FeCl_3_ for the oxidative C–C coupling reaction to form conjugated polymers. Nonetheless, to the best of the authors’ knowledge, the use of triflate salts has not been explored thus far in the synthesis of conjugated polymers via an all-solvent-free oCVD approach.

Herein, we explore triflate salts as alternative solid nonchlorinated oxidants to FeCl_3_ for the oCVD reaction to form fused metalloporphyrin conjugated polymer thin films and engineer their optoelectronic properties. Cobalt(II) 5,15-diphenyl porphyrin was selected as a model in this study due to its low redox potential (),^32^ in addition to the potential interest of cobalt(II) porphyrin conjugated polymers in catalysis and energy applications.^32,52−54^ Also, cobalt(II) porphyrins bearing F-containing aryl substituents, namely, 4-fluorophenyl, 4-trifluoromethylphenyl, and pentafluorophenyl groups, were prepared to gain a deeper understanding of the chemical structure and optoelectronic properties of the fused metalloporphyrin conjugated polymer thin films prepared using triflate salts and the significance of the inclusion of −CF_3_ groups.

## Results and Discussion

### oCVD Polymerization Reaction of Cobalt(II) 5,15-Diphenylporphyrin Using Triflates Salts

The simultaneous synthesis and deposition of cobalt(II) 5,15-diphenyl porphyrins conjugated polymers using triflate salts of iron(III) and copper(II), i.e., Fe(OTf)_3_ and Cu(OTf)_2_, respecively, as the oxidants—hereafter denoted as **pCoDPP-Fe(OTf)**_**3**_ and **pCoDPP-Cu(OTf)**_**2**_, respectively—were performed in a custom-built oCVD reactor described in detail elsewhere .^32^ It is worth noting here that silver(I) triflate, Ag(OTf), was also investigated but only yielded low polymerization efficiencies (Figure S1a of the SI) due to the low oxidation strength of the salt (*E*^0^(Ag^+^/Ag^0^) = 0.22 V). For the sake of comparison, cobalt(II) 5,15-diphenyl porphyrin conjugated polymer thin films were prepared by using FeCl_3_ as the oxidant, i.e., **pCoDPP-FeCl**_**3**_. In addition, a reference thin film was prepared from the sublimation of CoDPP in the absence of any oxidant (**sCoDPP**). Table S1 of the SI summarizes the experimental conditions used for the preparation of the thin films.

**Figure 1:**
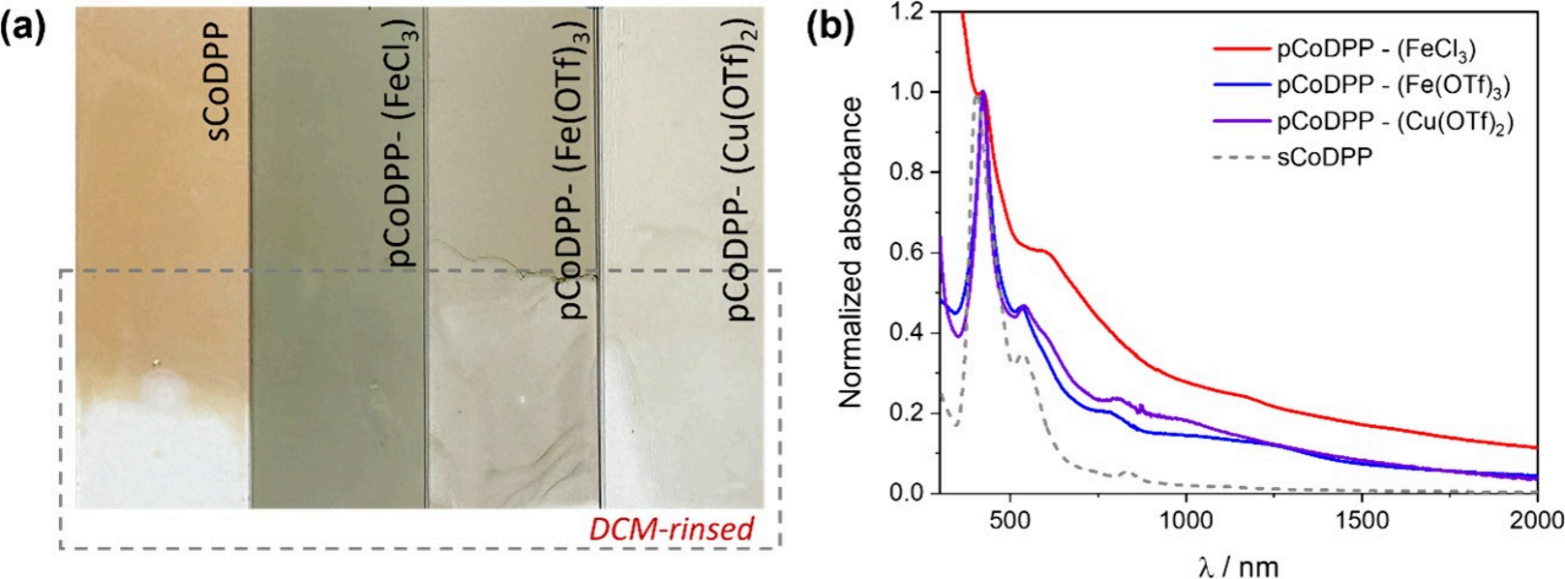
(a) Digital pictures of the as-deposited (top) and DCM-rinsed (bottom) reference thin film (left side, orangish) prepared from the sublimation of cobalt(II) 5,15-diphenyl porphyrin (CoDPP), **sCoDPP**, and the oCVD (right side, greenish) thin films prepared from CoDPP and the three different oxidants studied herein: **pCoDPP-(FeCl**_**3**_**)**, **pCoDPP-(Fe(OTf)**_**3**_**)**, and **pCoDPP-(Cu(OTf)**_**2**_**)**. The area inside the dashed box indicates a section of the thin films rinsed with dichloromethane (DCM). (b) UV–vis/NIR absorption spectra of the as-deposited reference sublimed and oCVD thin films.

As depicted in Figure 1a, the thin films prepared by the oCVD reaction of cobalt(II) 5,15-diphenyl porphyrin with the three different oxidants, i.e., **pCoDPP-FeCl**_**3**_ and **pCoDPP-Fe(OTf)**_**3**_, and **pCoDPP-Cu(OTf)**_**2**_, exhibited a notable color difference in comparison with the reference thin film obtained from the sublimation of the monomer in the absence of any oxidant agent, i.e., **sCoDPP**, pointing to the formation of fused metalloporphyrin conjugated polymers in the thin films prepared in the presence of any of the three studied oxidants. Specifically, the greenish-brownish coloration of the oCVD thin films is often indicative of a broadening and red shifting of the absorption bands. To gain further insights into the polymerization reaction, the thin films were rinsed with dichloromethane (DCM), an aprotic organic solvent able to dissolve monomers and small oligomers. As shown in Figure 1a, oCVD thin films synthesized using Fe(OTf)_3_ or Cu(OTf)_2_ as the oxidant partly dissolved in DCM, in contrast to the one prepared from FeCl_3_, suggesting a lower degree of polymerization when using triflate salts. It worth noting that chlorination is known to reduce the solubility of porphyrins.^55^

In agreement with the above observations, UV–vis/NIR absorption spectra in Figure 1b confirmed the broadening and red shifting of the absorption bands of the oCVD thin films compared to the reference sublimed thin film, confirming the formation of multiply fused metalloporphyrin conjugated polymers irrespective of the oxidant. Nonetheless, compared with the oCVD film from FeCl_3_, the absorption spectra of the thin films prepared from triflate salts as oxidants show less remarkable broadening and reduced intensity in the NIR region, pointing to a lower extension of polymerization or a lower degree of fusion. The UV–vis/NIR spectra of the thin films after rinsing with DCM (Figure S2b and c of the SI) also evidence the partial dissolution of **pCoDPP-(Fe(OTf)**_**3**_**)** and **pCoDPP-(Cu(OTf)**_**2**_**)**, suggesting the presence of short oligomers and monomers in these thin films. It is worth noting here that the oxidant/metalloporphyrin molar ratio during the oCVD reaction is significantly lower for the thin films prepared from triflate salts (11.2 and 10.9 for Fe(OTf)_3_ and Cu(Otf)_2_, respectively), in comparison with FeCl_3_ (88.4) due to the higher molecular weight of the triflate salts. Lower oxidant/metalloporphyrin molar ratios have been reported to yield lower dehydrogenative C–C coupling reaction rates.^41^

**Figure 2:**
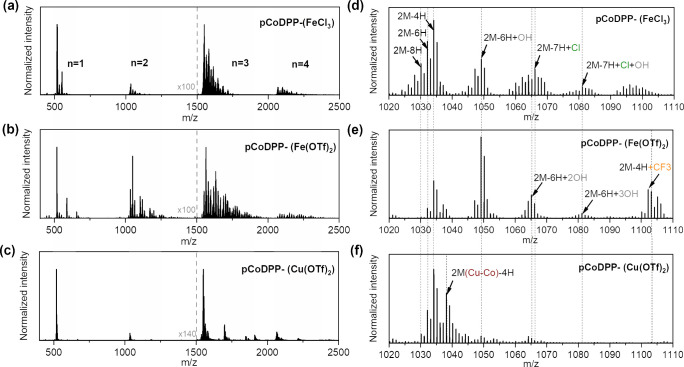
LDI-HRMS spectrum in the mass ranges (a–c) 400–3000 *m*/*z* and (d–f) 1020–1110 *m*/*z* (dimer region) of the thin films resulting from the oCVD reaction of CoDPP with the three different oxidants studied herein (**pCoDPP-(FeCl**_**3**_**)**, **pCoDPP-(Fe(OTf)**_**3**_**)**, and **pCoDPP-(Cu(OTf)**_**2**_**)**), showing the formation of oligomers up to *n* = 4 from intermolecular dehydrogenative C–C coupling (*n* = degree of oligomerization) and the occurrence of side reactions, i.e., −Cl, −CF_3_, or −OH inclusion, intramolecular dehydrogenative C–C coupling, demetalation, and remetalation.

Deeper insights into the reactivity of the investigated oxidants in oCVD were obtained from laser desorption/ionization high-resolution mass spectrometry (LDI-HRMS) analysis. As shown in Figure 2a, irrespective of the oxidant, all the oCVD thin films showed peak distributions associated with oligomers [(CoDPP)_*n*_ – (2H)_*m*_]^+^ with *m* ≥ *n*, indicating the fusion of the metalloporphyrin units by intermolecular dehydrogenative C–C coupling. Along with the dehydrogenative intermolecular C–C coupling (polymerization), intramolecular C–C coupling can also take place between free *ortho-*position of the aryl substituent and the *beta-*position of the porphyrin macrocycle.^36,56^ Previous studies have evidenced the role of intramolecular cyclization in the extension of the conjugated π-systems and the planarity of the fused metalloporphyrin tapes, with benefits on the electronic and optoelectronic properties.^56^

As shown in Figure 2d, the LDI-HRMS analysis in the dimer region of **pCoDPP-(FeCl**_**3**_**)** evidenced the loss of up to eight and ten hydrogen atoms, attributed to the formation of doubly and triply fused metalloporphyrin dimers, respectively, along with the occurrence of intramolecular cyclization between the phenyl groups and the porphyrin macrocycle.^32^ In contrast, the LDI-HRMS spectra in the dimer region of the thin films prepared using Fe(OTf)_3_ and Cu(OTf)_2_ as the oxidants evidence the loss of only four to six hydrogen atoms, pointing to a lower degree of fusion. This observation is consistent with the reduced extent of polymerization and the lower degree of fusion anticipated from the optical analysis (Figure 1**b**).

### Integration of −CF_3_ to the Fused Metalloporphyrin Conjugated Polymer Chains and Other Side Reactions

Besides the intermolecular C–C coupling reactions, other side reactions to direct fusing and polymerization can take place during the oCVD processing, such as the inclusion of chlorine/–CF_3_ groups, oxidation of the metal center, and demetalation/remetalation of the porphyrin macrocycle. As depicted in Figure 2b, the chlorination side reaction is clearly present in the oCVD thin film prepared with FeCl_3_, i.e., **pCoDPP-(FeCl**_**3**_**)**, giving rise to peak distributions shifted by 35 *m*/*z*, which corresponding to the exchange of hydrogen by chlorine atoms. Similar patterns are observed in the LDI-HRMS analysis of the trimer region provided in Figure S2. Chlorination of the fused metalloporphyrin conjugated polymer chains is also evidenced in the XPS analysis of **pCoDPP-(FeCl**_**3**_**)**. As shown in Figure S3, the XPS spectrum in the Cl 2p region reveals the presence of two chlorine environments: a main contribution at lower binding energies (Cl 2p_3/2_ = 198.7 eV and Cl 2p_1/2_ = 200.3 eV) associated with metal chloride due to the presence of unreacted FeCl_3_ or FeCl_3_ byproducts (e.g., FeCl_2_), and a contribution at higher binding energies (Cl 2p_3/2_ = 200.6 eV and Cl 2p_1/2_ = 202.2 eV) related to organic chlorine bonded to the porphyrin macrocycle and its phenyl substituents.^32,36,37^

**Figure 3:**
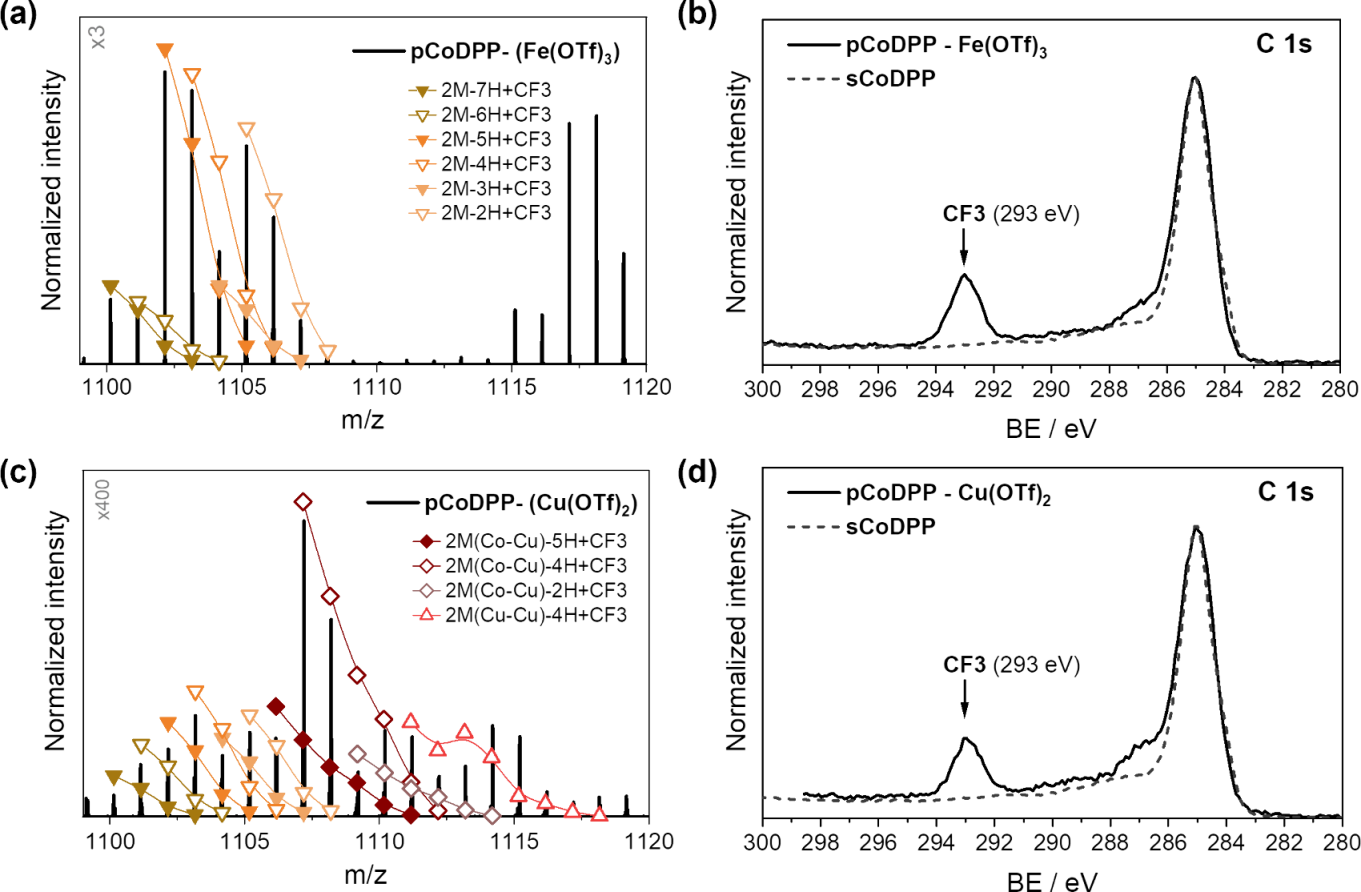
(a–c) LDI-HRMS spectra in the dimer region (mass range 1099–1120 *m*/*z*) and (b–d) XPS spectra of the C 1s core level of **pCoDPP-(Fe(OTf)**_**3**_**)** (a and b) and **pCoDPP-(Cu(OTf)**_**2**_**)** (c and d) thin films. In (a) and (c), the simulated isotopic patterns for [(CoDPP)_2_ – (2H)_*m*_ + CF_3_ – H]^+^ and [(CoDPP-CuDPP) – (2H)_*m*_ + CF_3_ – H]^+^, as well as [(CoDPP)_2_ – -(2H)_*m*_ + CF_3_]^+^ and [(CoDPP-CuDPP) – (2H)_*m*_ + CF_3_]^+^, are reported for ease of comparison. For simplicity, the [(CoDPP)_2_ – (2H)_*m*_ + CF_3_ – H]^+^ species are labeled in the figures as 2M-(2*m*+1)H+CF_3_ (filled symbols), while [(CoDPP)_2_ -(2H)_*m*_ + CF_3_]^+^ (open symbols, corresponding to the adduct) are labeled as 2M-2*m*H+CF_3_.

On the other hand, the presence of −CF_3_ groups is clearly evidenced in the LDI-HRMS spectra in the dimer and trimer regions (Figure 2e and Figure S2e) of the thin film prepared using Fe(OTf)_3_, i.e., **pCoDPP-(Fe(OTf)**_**3**_**)**. Moreover, the addition of −CF_3_ groups to the porphyrin macrocycle and/or the phenyl substituents is confirmed through comparison with the simulated isotopic patterns (represented with filled symbols in Figure 3a) in the dimer region of the LDI-HRMS spectra of **pCoDPP-(Fe(OTf)**_**3**_**)**. It is worth noting that the inclusion of −CF_3_ groups appears both from exchange with hydrogen atoms ([(CoDPP)_2_ – (2H)_*m*_ + CF_3_]^+^ species represented in Figure 3a as 2M-(*m*2 + 1)H+CF_3_ (filled symbols)) taking place during the oCVD process and from the formation of adducts ([(CoDPP)_2_ – (2H)_*m*_ + CF_3_]^+^ species represented in Figure 3a as 2M-2*m*H+CF_3_ (empty symbols), the latter formed in source during the analysis. In addition, the presence of CF_3_-containing species is also evidenced in the XPS spectrum in the C 1s core region, depicted in Figure 3b, showing a signal at 293.0 eV characteristic of −CF_3_ groups either linked to the porphyrin macrocycle and/or the phenyl substituents or from unreacted oxidant and byproducts residuals at the thin film surface.

In contrast, the inclusion of −CF_3_ groups is less perceptible in the LDI-HRMS spectra of **pCoDPP-(Cu(OTf)**_**2**_**)**, as depicted in Figure 2f and Figure S2f. Notably, Figure 2f shows an intense peak signal at 1038.16 *m*/*z* in the LDI-HRMS spectrum of **pCoDPP-(Cu(OTf)**_**2**_**)** corresponding to [(CoDPP-CuDPP) – (2H)_2_]^+^ species (simplified as 2M(Co-Cu)-4H), clearly evidencing the occurrence of porphyrin macrocycle demetalation and subsequent remetalation with copper(II) ions from the oxidant. Further confirmation is depicted in Figure S5 through comparison with the simulated isotopic pattern corresponding to homometallic (CoDPP-CoDPP) and heterometallic (CoDPP-CuDPP) dimers. Likewise, comparison of the experimental spectra with the isotopic patterns in the mass range 1100–1120 *m*/*z* (Figure 3c) reveals the coexistence of CF_3_-containing homometallated (CoDPP-CoDPP and CuDPP-CuDPP) and heterometallated (CoDPP-CuDPP) dimers. Although peaks corresponding to the integration of −CF_3_ to the porphyrin macrocycle and/or the phenyl substituents involving hydrogen atom exchange are detected (simulated patterns represented with filled symbols in Figure 3c), a contribution related to noncovalent −CF_3_ inclusion (simulated patterns represented with empty symbols in Figure 3c) occurring during the sample’s analysis is also evidenced. The XPS analysis of the C 1s core level region of the sample prepared with Cu(OTf)_2_ as the oxidant (Figure 3d) displays the characteristic peak at 293.0 eV, further confirming the presence of -CF_3_ groups, although like in **pCoDPP-(Fe(OTf)**_**3**_**)** the contribution of unreacted oxidant and remnant byproducts to this signal should not be discarded. Further discussion on the fate of the oxidant used during the oCVD process, including reaction and sublimation/thermal decomposition byproducts, and the possible mechanism for the inclusion of chlorine/–CF_3_ groups in the porphyrin macrocycle and the aromatic substituent are included in the Supporting Information.

**Figure 4:**
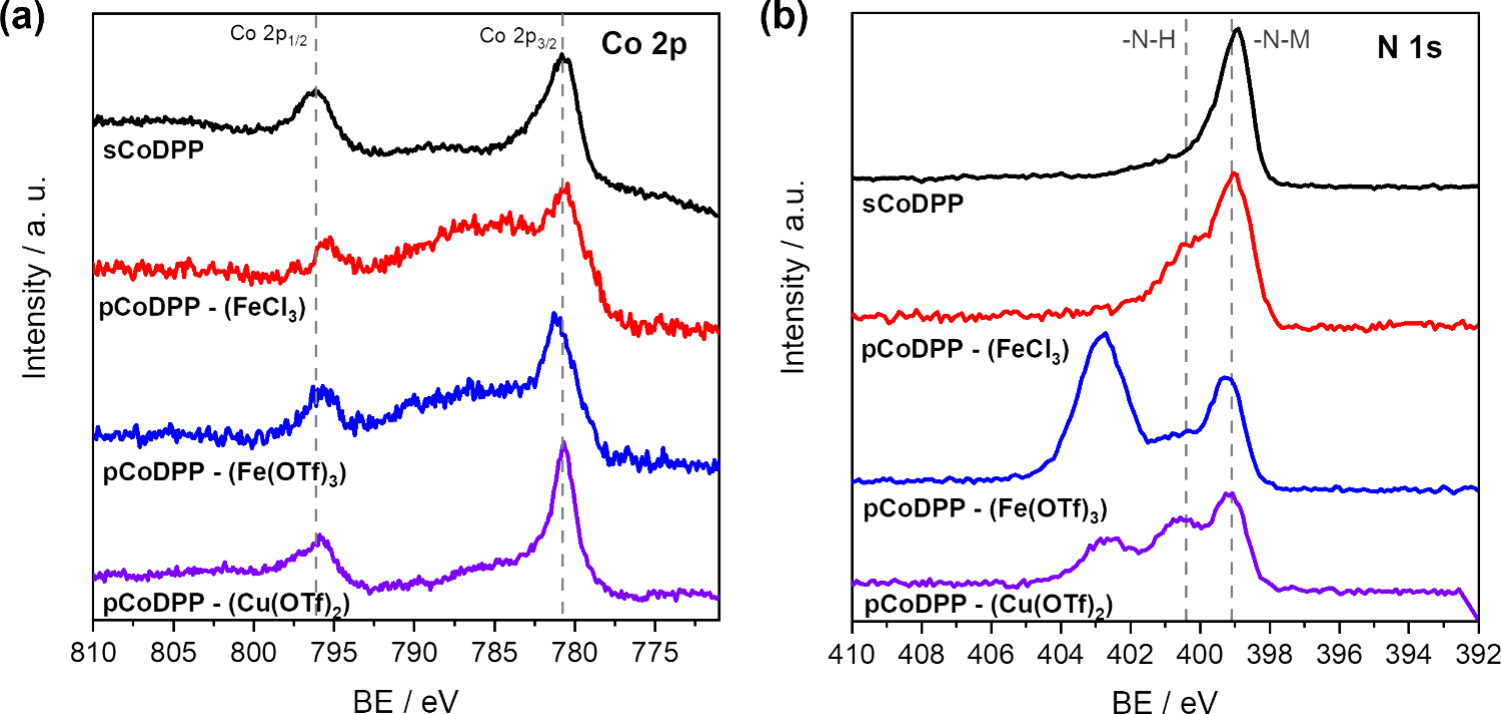
XPS spectra of the (a) Co 2p and (b) N 1s core level regions of the reference thin film prepared from the sublimation of CoDPP, **sCoDPP**, and the oCVD thin films prepared from CoDPP and the three different oxidants studied herein (**pCoDPP-(FeCl**_**3**_**)**, **pCoDPP-(Fe(OTf)**_**3**_**)**, and **pCoDPP-(Cu(OTf)**_**2**_**)**).

It is worth noting that the LDI-HRMS spectrum in the dimer region of **pCoDPP-(Fe(OTf)**_**3**_**)** is dominated by the contribution of [(CoDPP)_2_ – (2H)_3_ + OH]^+^ species (in Figure 2e simplified as 2M-6H+OH). The inclusion of −OH both with and without exchange with one hydrogen atom—the former case pointing to the oxidation of the metal center (Co^2+^ to Co^3+^)—is evidenced by comparison with isotopic patterns in the mass range 1045–1053 *m*/*z* (Figure S4). Previous studies from our group reported the isomeric valence nature of the cobalt porphyrin radical cations, [Co(II)DPP]^+^ and [Co(III)DPP]^+^, formed during the oCVD process using FeCl_3_.^32,37,57^ As shown in Figure 4a, a main contribution of the Co 2p_3/2_ signal is observed in the sublimed reference coatings at ca. 780.7 eV, characteristic of cobalt(II) porphyrins.^32,37,57^ However, the presence of both Co^2+^ and Co^3+^ oxidation states is evidenced in the XPS spectra of **pCoDPP-(FeCl**_**3**_**)** and **pCoDPP-(Fe(OTf)**_**3**_**)** as the broadening and slight shift of the Co 2p_3/2_ peak toward higher binding energies. In contrast, such behavior is not clearly depicted for **pCoDPP-(Cu(OTf)**_**2**_**)**. This observation is consistent with the lower intensity of the characteristic peaks related to the inclusion of −OH in the LDI-HRMS spectra (Figure 2f) and can be related to the dominant demetalation/remetalation of the porphyrin macrocycle with copper(II) from the oxidant, as previously evidenced.

On the other hand, the XPS spectra in the N 1s region show for all thin films a main peak at ca. 399.1 eV, characteristic of the pyrrolic nitrogen (M–H) in metalloporphyrins (Figure 4b).^32,37^ Notably, the XPS spectrum of the N 1s core level region of **pCoDPP-(FeCl**_**3**_**)** exhibits a peak contribution at higher binding energy (ca. 400.4 eV) that points to the presence of amino groups (N–H), consistent with the presence of both Co(II) and Co(III) porphyrins.^37,57^ In addition, the N1s XPS spectra of **pCoDPP-(Fe(OTf)**_**3**_**)** and **pCoDPP-(Cu(OTf)**_**2**_**)** exhibit a contribution at higher binding energy (ca. 402.7 eV), pointing to the presence of nitrogen atoms with increased positive charge. Such an observation can be attributed to the noncovalent interaction of the porphyrin with −CF_3_ adducts formed during the sample analysis.

### Effect of the Oxidant on the Energy Levels of Fused Co(II) Porphyrin Conjugated Polymers

**Figure 5:**
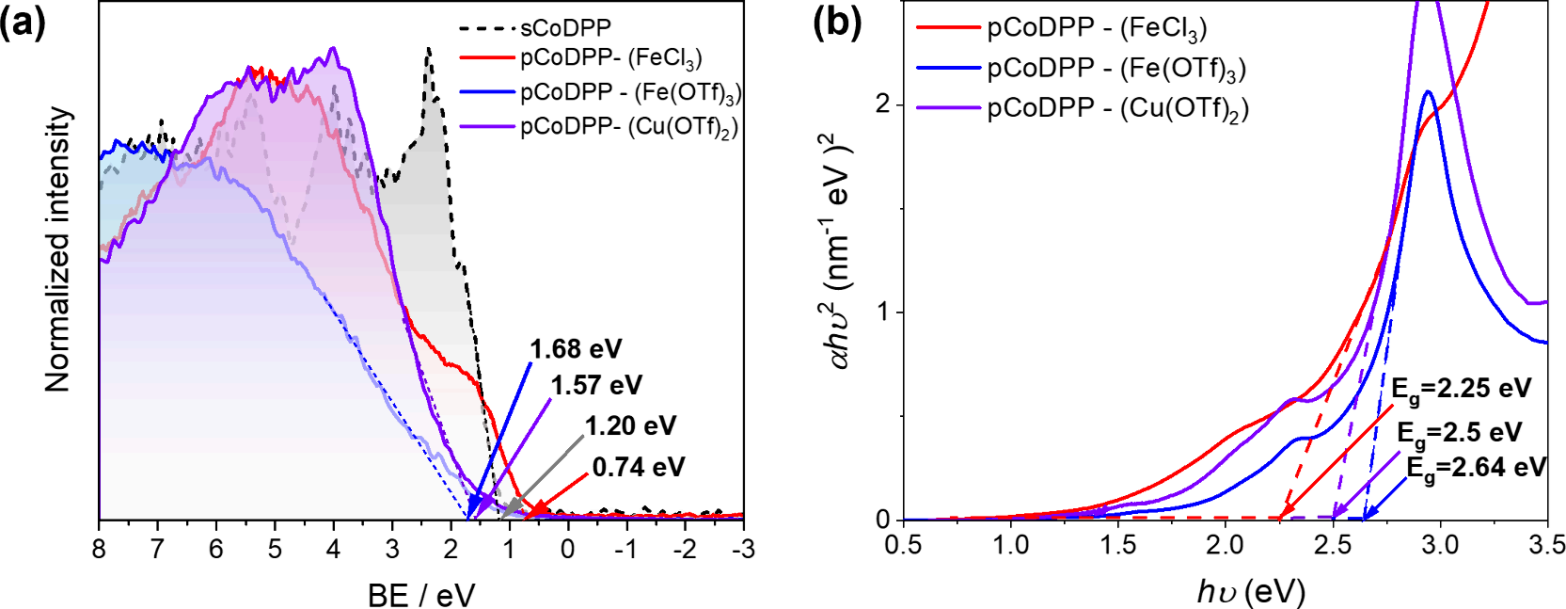
(a) Valence band edge XPS spectra of the reference sublimed and oCVD thin films prepared from CoDPP and (b) Tauc plot obtained from the absorption spectra of the oCVD thin films prepared from CoDPP and the three different oxidants studied herein.

The valence band maxima (VBM) of the oCVD and reference sublimed thin films were estimated from the valence band edge region of the XPS spectra (Figure 5a). In accordance with previous reports, the oCVD thin film prepared from FeCl_3_ as the oxidant, i.e., **pCoDPP-(FeCl**_**3**_**)**, depicts a notable shift of the VBM toward lower values (VBM = 0.74 eV) compared to the reference sublimed thin film **sCoDPP** (VBM = 1.20 eV). Such an observation is consistent with the intermolecular dehydrogenative C–-C coupling of the metalloporphyrin units and an extension of the conjugated π-electron system. Indeed, the extension of the π-conjugated system is known to raise the energy of the highest occupied molecular orbital (HOMO) and lead to a low excitation gap in fused metalloporphyrin tapes.^58^ Surprisingly, the oCVD thin films prepared from triflate salts, i.e., **pCoDPP-(Fe(OTf)**_**3**_**)** and **pCoDPP-(Cu(OTf)**_**2**_**)**, show a shift of their VBM to higher binding energies. This observation is consistent with the aforementioned integration of −CF_3_ groups as a result of the use of the triflate salts as the oxidant during the oCVD reaction. Although fused metalloporphyrin tapes are formed, as evidenced from the UV–vis/NIR and LDI-HRMS observations, the integration of −CF_3_ groups appears to play a significant role in the position of VBM. Indeed, the strong electron-withdrawing properties of fluorine are well-known to deepen the valence band (or HOMO) of conjugated polymers.^59−61^ Consistently, a slightly higher shift of the VBM is observed for the oCVD thin film prepared from Fe(OTf)_3_, (**pCoDPP-(Fe(OTf)**_**3**_, VBM = 1.68 eV), which integrates a larger number of −CF_3_ groups into the fused metalloporphyrin tapes, than for the oCVD thin film prepared from Cu(OTf)_2_ (**pCoDPP-(Cu(OTf)**_**2**_, VBM = 1.57 eV), which shows minor integration of −CF_3_ groups with respect to **pCoDPP-(Fe(OTf)**_**3**_.

Interestingly, the band gap energies (*E*_g_)—estimated from the Tauc plot obtained from the absorbance spectra of the oCVD thin films, as shown in Figure 5b—display rather close values for all the oCVD thin films, consistent with the extension of the π-conjugated system from all the studied oxidants. This observation notably confirms that the lower extension of polymerization suspected for the oCVD thin films prepared using triflate salts cannot exclusively account for the opposite shift of the VBM with respect to the sublimed thin film and, more generally, to the resulting optoelectronic properties. Indeed, one can note that **pCoDPP-(Cu(OTf)**_**2**_**)** exhibits a lower *E*_g_ value than **pCoDPP-(Fe(OTf)**_**3**_**)**, while both triflate salts demonstrate a similar extent of polymerization. However, as pointed out from LDI-HRMS analysis, using Cu(OTf)_2_ as the oxidant promotes the demetalation of the porphyrin center and further remetalation with copper(II) from the oxidant, leading to Co–Cu heterometallic conjugated polymers. Interestingly, a previous study from our group showed that the **p(CuDPP-co-CoDPP)** heterometallic conjugated polymer displays a significantly reduced *E*_g_ as a result as internal charge transfer (ICT) characteristic of D–A copolymers.^62−64^ Moreover, it was also demonstrated that heterometalated porphyrin conjugated polymers can achieve higher electrical conductivities.^62^ Therefore, careful selection of the oxidant allows control of not only the extension of polymerization but also the side reactions such as the oxidant’s counterion inclusion and demetalation/metalation of the porphyrin macrocycle, offering a convenient strategy for engineering the optoelectronic properties of conjugated polymer thin films.

### Impact of −-CF_3_ Addition during the oCVD Reaction vs Polymerization of F-Containing Cobalt(II) 5,15-Disubstituted Porphyrins

**Scheme 2:**
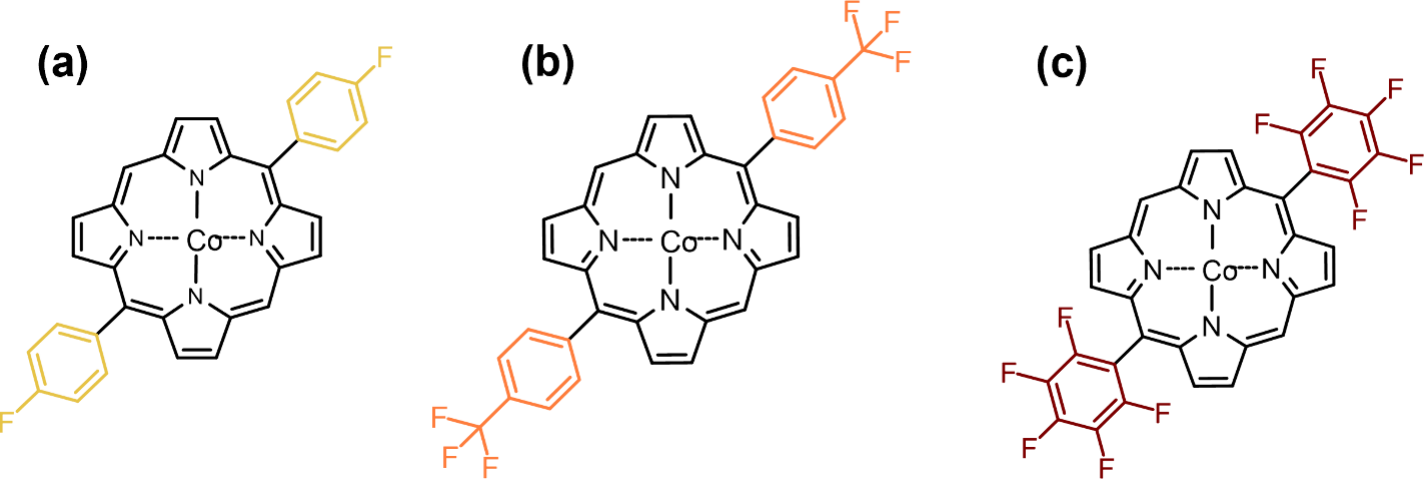
Molecular Structures of the 5,15-Disubstituted Cobalt(II) Porphyrins with F-Containing Aryl Groups (a) 4-Fluorophenyl (4-FP), (b) 4-trifluoromethylphenyl (4-CF_3_P), and (c) pentafluorophenyl (PFP).

To better understand the effect and benefit of the inclusion of −CF_3_ groups during the synthesis and deposition of fused cobalt(II) porphyrin conjugated polymers when using triflate salts in comparison with the oCVD polymerization of porphyrins already bearing fluorinated substituents, the oCVD reactions of three F-containing 5,15-disubstituted cobalt(II) porphyrins, represented in Scheme 2, were performed using FeCl_3_ as the oxidant. Table S1 of the SI summarizes the detailed deposition conditions (amount and sublimation temperatures of the monomer and the oxidant) of the oCVD thin films. In addition, thin films from the sublimed monomers, i.e., in the absence of any oxidant agent, were also prepared as references. The resulting oCVD and reference sublimed thin films are denoted as **pCoDXP-(FeCl**_**3**_**)** and **sCoDXP**, respectively, with X = P (phenyl), 4-CF_3_PP (4-trifluoromethylphenyl), 4-FP (4-fluorophenyl), and PFP (pentafluorophenyl).

**Figure 6:**
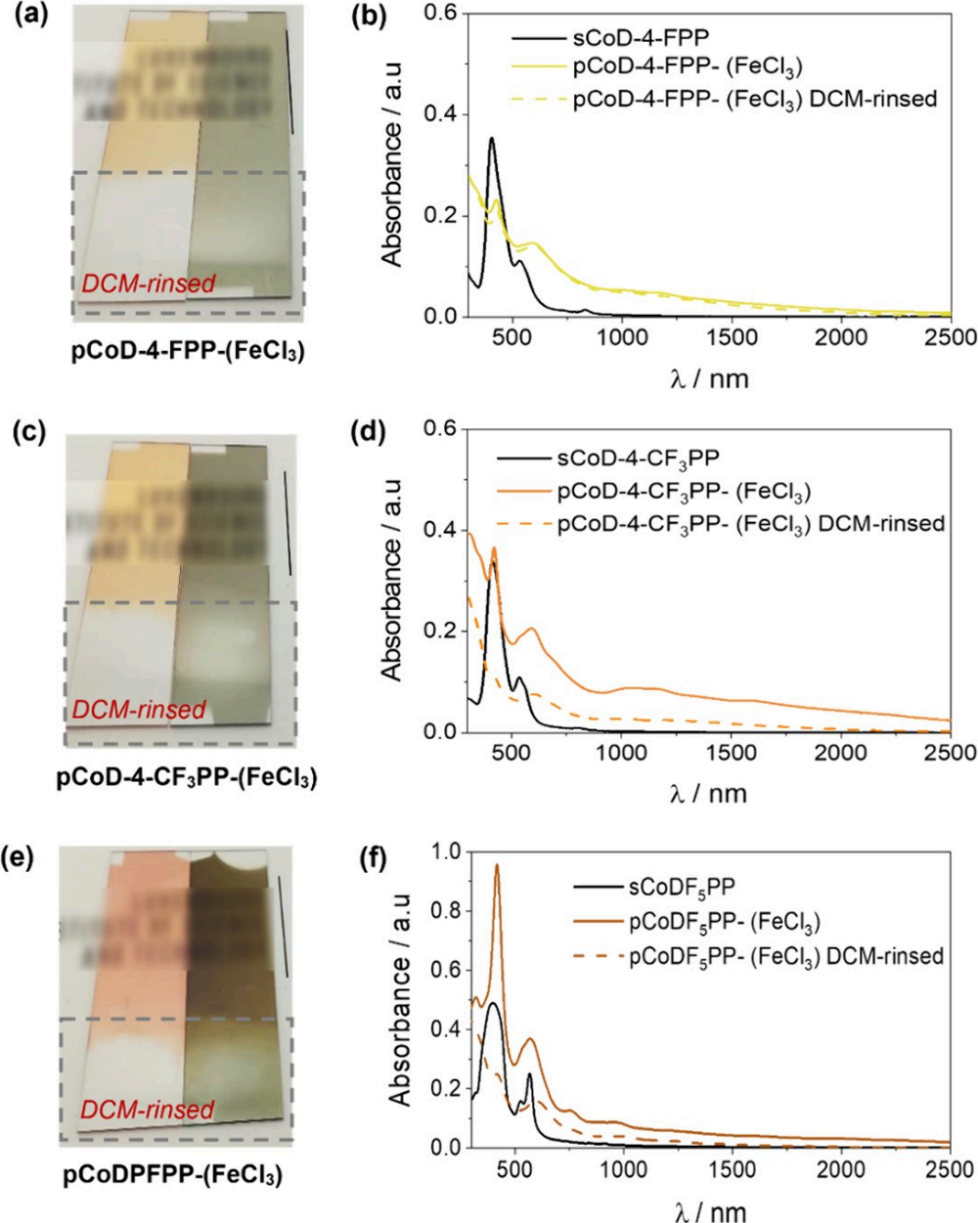
(a, c, and e) Digital pictures of the as-deposited (top) and DCM-rinsed (bottom) reference sublimed (left side, orangish) and oCVD (right side, greenish) thin films prepared from F-containing cobalt(II) porphyrins, i.e., CoD-4-FPP, CoDP-4-CF_3_PP, and CoDPFPP. The DCM-rinsed area is marked inside the dash-lined box. (b, d, and f) UV–vis/NIR absorbance spectra of the as-deposited (straight lines) and DCM-rinsed (dashed lines) reference sublimed (black lines) and oCVD (colored lines) thin films prepared from the F-containing cobalt(II) porphyrins.

As depicted in Figure 6, all the thin films obtained from the oCVD reaction of fluorine-containing 5,15-disubstituted cobalt(II) porphyrins (greenish) exhibited a notable color difference compared to the reference thin films (orangish), i.e., those obtained from the sublimation of the porphyrin monomers in absence of the oxidant agent. Such a color difference, similar to the one reported above, is indicative of the formation of fused metalloporphyrin conjugated polymers.

UV–vis/NIR analysis of as-deposited **pCoD-4-FPP-(FeCl**_**3**_**)** and **pCoD-4-CF**_**3**_**PP-(FeCl**_**3**_**)** showed broadened Soret and Q-bands and absorption in the NIR spectral region, as depicted in Figure 6b and d. Such enhanced absorption is indicative of the formation of multiply fused metalloporphyrin tapes.^35,65^ The spectra of **pCoD-4-FPP-(FeCl**_**3**_**)** remain unaltered after rinsing with dichloromethane, confirming the formation of highly conjugated and extended oligomers. However, **pCoD-4-CF**_**3**_**PP-(FeCl**_**3**_**)** partially dissolves in DCM, as depicted in Figure 5c, suggesting the formation of shorter oligomers. Finally, the absorbance spectra obtained for **pCoDPFPP-(FeCl**_**3**_**)** display rather similar features compared to its reference sublimed thin film, i.e., **sCoDPFPP**, displaying no significant shift of the Soret and Q-bands and moderate absorption in the NIR region (Figure 6f). This observation points to CoDPFPP failing to yield extended fused metalloporphyrin conjugated polymer chains.

**Figure 7:**
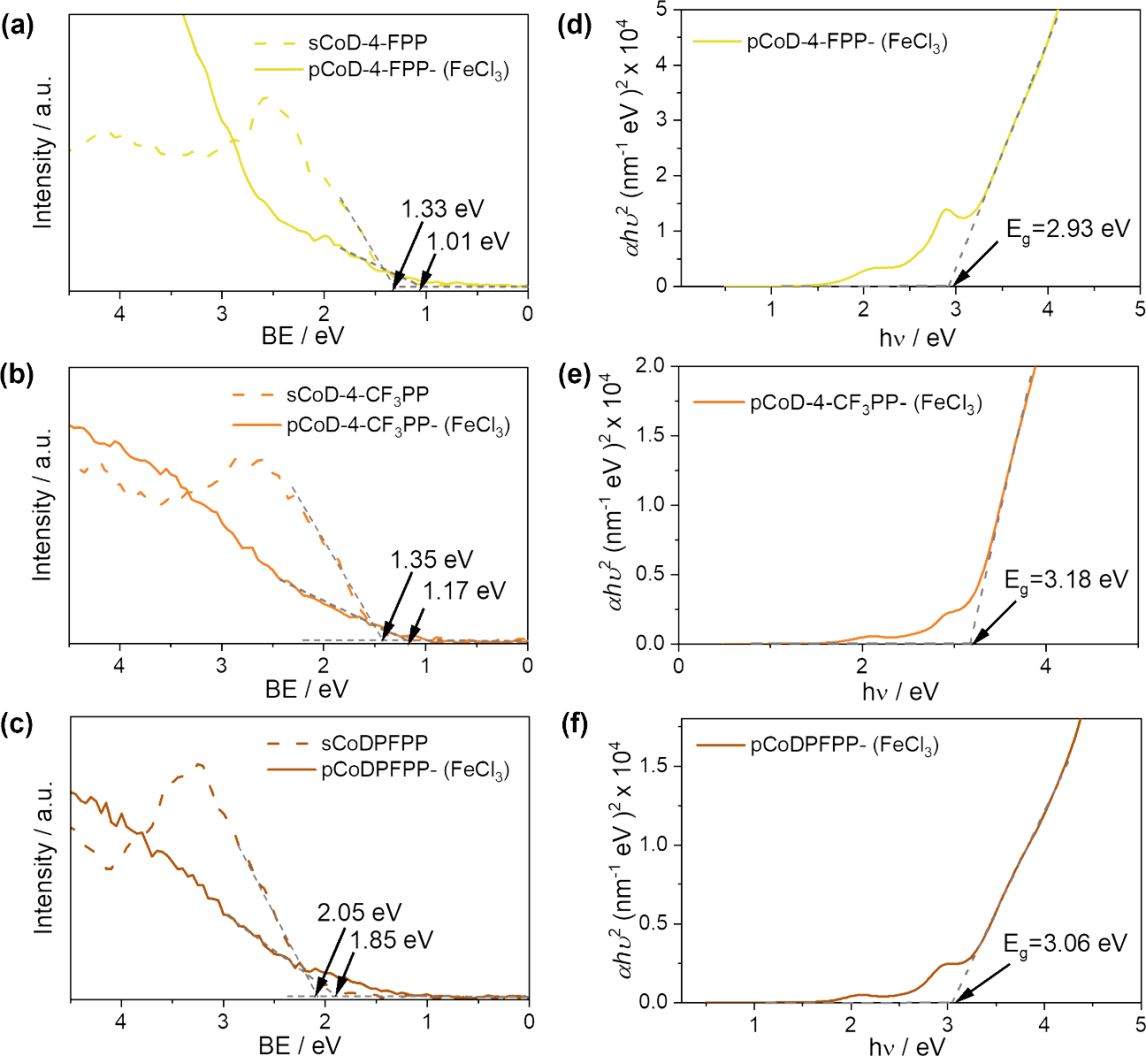
(a–c) Valence band (VB) edge XPS spectra of the reference sublimed (dashed lines) and oCVD (straight lines) thin films prepared from F-containing cobalt(II) porphyrins, i.e., CoD-4-FPP, CoDP-4-CF_3_PP, and CoDPFPP. The evaluated valence band maxima (VBM) are indicated by arrows. (d–f) Tauc plots derived from the absorption spectra of the oCVD thin films. The estimated energy band gaps (*E*_g_) are indicated by arrows.

LDI-HRMS confirms the occurrence of intermolecular dehydrogenative C–C coupling reaction of CoD-4-FPP and CoD-4CF_3_PP in oCVD, along with possible intramolecular dehydrogenative C–C coupling (cyclization) (Figure S6). In addition, the occurrence of chlorination and −OH inclusion side reactions were also evidenced in all the oCVD thin films, regardless of the aryl substituent attached to the porphyrin macrocycle. The VBM was estimated from valence band (VB) edge XPS spectra shown in Figure 7a. In all cases, the VBM shifts toward lower values, with the lowest shift observed for **pCoDPFPP-(FeCl**_**3**_**)**, in agreement with the lower polymerization efficiency previously suggested by UV–vis/NIR and LDI-HRMS analyses.

**Figure 8:**
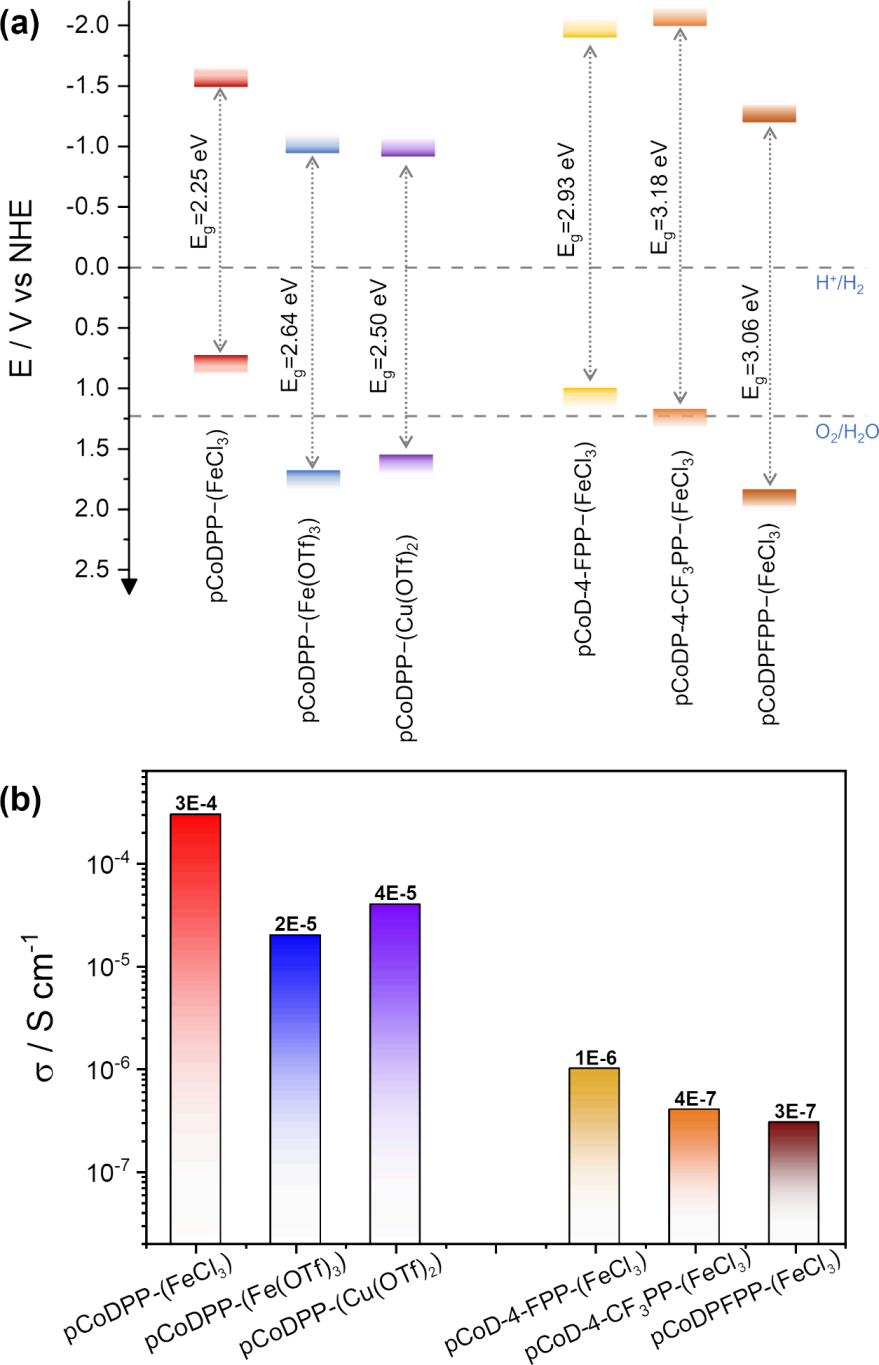
(a) Energy band diagram and (b) linear conductivity of the oCVD thin films prepared from (left) CoDPP and the three different oxidants studied herein, i.e., **pCoDPP-(FeCl**_**3**_**)**, **pCoDPP-(Fe(OTf)**_**3**_**)**, and **pCoDPP-(Cu(OTf)**_**2**_**)**, and (right) CoD-4-FPP, CoDP-4-CF_3_PP, and CoDPFPP using FeCl_3_ as the oxidant, i.e., **pCoD-4-FPP-(FeCl**_**3**_**)**, **pCoDP-4-CF**_**3**_**PP-(FeCl**_**3**_**)**, and **pCoDPFPP-(FeCl**_**3**_**)**. The redox potentials for proton reduction and water oxidation reactions in acidic media are presented as references.

From the VBM values obtained by XPS, and the band gap (*E*_g_) calculated from the Tauc plots derived from the UV–vis/NIR spectroscopy measurements (Figure 7d–f), the energy band arrangements of all the oCVD thin films studied in this work were estimated (Figure 8a). In addition, lateral conductivity measurements were performed on the oCVD thin films deposited on chips patterned with interdigitated electrodes (Figure S7). As shown in Figure 8a, **pCoDPP-(Fe(OTf)**_**3**_**)**, **pCoDPP-(Cu(OTf)**_**2**_**)**, and **pCoDPFPP-(FeCl**_**3**_**)** display deeper VBM positions given the presence of fluorine atoms attached to the porphyrin macrocycles or/and their aryl substituents. Moreover, **pCoDPFPP-(FeCl**_**3**_**)** displays the lowest conductivity, followed by **pCoD-4-CF**_**3**_**PP-(FeCl**_**3**_**)** and **pCoD-4-FPP-(FeCl**_**3**_**)**, which is consistent with the lower polymerization efficiency suggested by UV–vis/NIR and LDI-HRMS analyses of these films with respect to those prepared using the triflate salts. Indeed, porphyrin substituents possessing electron-withdrawing character were previously demonstrated to be responsible for a lower extent of intermolecular and intramolecular dehydrogenative C–C couplings in fused metalloporphyrin tapes.^39^ Thus, despite enabling a lowering of the VBM position, the use of porphyrin monomers with fluorine-containing groups (i.e., the smallest electron-withdrawing atom) does not provide a convenient strategy for the formation of extended fused metalloporphyrin conjugated polymers.

It is worth mentioning that the lower conductivity of the cobalt(II) 5,15-diphenyl porphyrin thin films prepared using the triflate salts correlates with the lower extension of polymerization of these films with respect to **pCoDPP-(FeCl**_**3**_**)** evidenced in the UV–vis/NIR analysis due to the lower oxidation potential of the triflate salts and the lower oxidant/metalloporphyrin molar ratio during the oCVD reaction. However, as discussed in the previous section, the lower extension of polymerization when using triflate salts cannot exclusively account for the resulting electric and optoelectronic properties. Indeed, the integration of −CF_3_ groups plays a significant role in the position of the VBM. Specifically, the addition of −CF_3_ groups in **pCoDPP-(Fe(OTf)**_**3**_**)** and **pCoDPP-(Cu(OTf)**_**2**_**)** allows a higher VBM than **pCoDPFPP-(FeCl**_**3**_**)**. One can note that VBM values when using the triflates salts remain below the water oxidation potential (1.23 V vs NHE), while the conduction band minimum (CBM) is set above the hydrogen evolution potential (0 V vs NHE). This result is convenient for the potential implementation of these fused metalloporphyrin conjugated polymers for hydrogen production schemes. Therefore, the oxidative polymerization of metalloporphyrins using triflate salts in oCVD appears as an attractive strategy for the significant integration of electron-withdrawing −CF_3_ groups in the fused metalloporphyrin tapes to tune the VBM position while ensuring both the inter- and intramolecular dehydrogenative C–C coupling of metalloporphyrins.

Interestingly, using triflate salts also results in conjugated polymer thin films with lower band gaps (2.50–2.64 eV) in comparison with the conjugated polymer thin films prepared from F-containing cobalt(II) porphyrins and FeCl_3_ (2.93–3.18 eV), which makes the use of triflate salts a more attractive approach for the preparation of fused metalloporphyrin conjugated polymers suitable for photon-absorption and solar energy harvesting devices. The strategy developed herein is not specific to fused metalloporphyrin conjugated polymers and could be readily implemented for manipulating the position of the frontier energy levels of other aromatic compounds and conjugated polymers, fostering their engineering for the broad range of applications where they are currently investigated. Indeed, the use of triflate salts in oCVD, enabling the simultaneous intermolecular dehydrogenative C–C coupling of aromatic compounds and their trifluoromethylation, suppresses the need for complicated and low-yield pre- or postfunctionalization solution-based steps involving trifluoromethylating reagents^66^ such as methyl 2,2-difluoro-2-(chlorsulfonyl)acetate,^67^*S*-(trifluoromethyl)-3,7-dinitrobenzothiophene trifluoro-methanesulfonate,^68^ trifluoroiodomethane,^69^ triflyl chloride,^70^ sodium triflinate,^71^ 2,8-diflouro-*S*-(trifluoromethyl)dibenzothiophenium salt,^72^ or 1-trifluoromethyl-1,2-benziodoxol-3(1*H*)-one.^73^ Moreover, trifluoromethylation is broadly used to manipulate the optical and electrochemical properties of conjugated polymers. Specifically, the integration of electron-withdrawing groups, including trifluoromethyl, is commonly used to deepen the valence band of conjugated polymers^59−61^ and enhance their functional properties, notably yielding highly efficient polymer-based solar cells.^74−76^

## Conclusions

For the first time, Fe(OTf)_3_ and Cu(OTf)_2_ were explored as alternative solid nonchlorinated oxidants for the formation of conjugated polymer thin films in oCVD. Interestingly, the significant effect of the oxidant used in oCVD on the formation of conjugated polymers and the occurrence of side reactions, such as oxidation of the metal center, inclusion of oxidant’s counterion, and demetalation/metalation of the porphyrin macrocycle, and the resulting optoelectronic properties was evidenced. Importantly, although the studied triflate salts enable the formation of fused metalloporphyrin tapes and, therefore, the extension of the conjugated π-electron system, as evidenced from the UV–vis/NIR and LDI-HRMS analysis, the integration of −CF_3_ groups appears to play a more significant role in the position of VBM of the resulting thin films. Indeed, the strong electron-withdrawing properties of fluorine are well-known to deepen the valence band (or HOMO) energy level of conjugated polymers. Interestingly, the occurrence of inter- and intramolecular C–C coupling in **pCoDPP-(Fe(OTf)**_**3**_**)** and **pCoDPP-(Cu(OTf)**_**2**_**)** affords a higher extension of the π-electron system with respect to F-containing porphyrins and therefore a lower *E*_g_ and higher conductivity than porphyrins with F-containing aryl substituents. The controlled polymerization and side reactions provided by using the triflate salts, leading to modified optoelectronic properties, opens a new horizon for the engineering of conjugated polymers with the desired characteristics for catalysis and solar energy harvesting applications.

## Experimental Section

### Reactants

Cobalt(II) 5,15-disubstituted porphyrins were obtained from PorphyChem (98%) and were used without further purification. Iron(III) chloride (FeCl_3_, 97%), iron(III) trifluoromethane sulfonate (Fe(OTf)_3_, 90%) and copper(II) trifluoromethane sulfonate (Cu(OTf)_2_, 98%) were used as oxidants in the oCVD process. All the oxidants were obtained from Sigma-Aldrich and used without further purification. Dichloromethane (HPLC grade > 99.8%, SupraSolv) was used as a solvent for optical characterizations.

### Oxidative Chemical Vapor Deposition (oCVD)

The oCVD reaction was performed in a custom-built oCVD reactor, as represented in Scheme S1 of the SI. Glass microscope slides (Menzel-Gläser Superfrost), silicon wafers (Siegert Wafer), and interdigitated chips (OFET Gen4, Fraunhofer) were used as substrates. Prior to deposition, all the substrates were cleaned with absolute ethanol (99.98%, VWR chemicals) and dried with nitrogen gas. The substrate holder was kept at 150 °C for all the depositions. The pressure inside the oCVD reactor was kept at 10^–3^ mbar under an argon (99.999%, Air Liquide) atmosphere. The deposition time was set to 30 min for all experiments. Specific sublimation conditions (i.e., consumed amount and temperature) of the porphyrins and oxidants investigated are provided in Table S1 of the SI. Additionally, reference sublimed porphyrin monomer thin films were obtained under the same conditions without supplying any oxidant.

### Thin Film characterization

The ultraviolet–visible–near-infrared (UV–vis/NIR) spectra of the sublimed and oCVD films deposited on glass slides were recorded with a PerkinElmer Lambda 1050 spectrometer in the transmission (*T*) mode in the 300–2500 nm wavelength interval. The absorbance (*A*) was calculated as *A* = −log(*T*). From the absorbance spectra, the direct optical band gap (*E*_g_) of the fused metalloporphyrins was estimated through the Tauc plot as , where α is the absorbance coefficient, *n* = 1/2 for direct transitions, *h* is Planck’s constant, and ν is the wavelength number. The absorbance coefficient was calculated as α = ln(10)*A*/*l*, where *l* is the film’s thickness. The film’s thickness was determined with an alpha-SE spectroscopic ellipsometer. The spectroscopic ellipsometry measurements were acquired at three angles (65°, 70°, and 75°), in the wavelength range of 500–900 nm. The data were fit to a Cauchy model. Additionally, the films were rinsed with dichloromethane (HPLC grade >99.8%, SupraSolv) to record the absorbance spectra of the solvent-soluble phase. Transparent quartz cuvettes of 3.5 mL capacity and 1 cm light path were used for the measurements of the solvent-soluble fraction. The rinsed glass slides were also analyzed for comparison with the as-deposited thin films.

Laser desorption/ionization high-resolution mass spectrometry (LDI-HRMS) measurements were performed using an AP-MALDI UHR ion source (MassTech, Inc.) coupled to an LTQ/Orbitrap Elite instrument (ThermoScientific). In-source fragmentation (*E* = 70 V) was used to prevent the formation of clusters. The measurements were performed on Si wafer substrates coated with either the sublimed porphyrin monomers or oCVD thin films, which were directly placed on the sample holder. LDI-HRMS measurements and analyses of metalloporphyrin-based thin films have been described in detail elsewhere.^36,37,41^ X-ray photoelectron spectroscopy (XPS) measurements were performed with a Kratos Axis Ultra DLD instrument using a monochromatic Al K_α_ X-ray source of energy 1486.6 eV at 105 W power. Charge calibration was accomplished by fixing the binding energy of carbon (C 1s) to 285.0 eV. Secondary ion mass spectrometry (SIMS) analyses were performed on a CAMECA NanoSIMS 50 instrument using a Cs^+^ primary ion beam with an impact energy of 16 keV and a current of 1.5 pA on the sample surface.

The conductivity of the thin films was evaluated by using a microprobe station (Cascade Microtech, PM8). The geometry of the interdigitated electrode channel was 2.5 μm (length) × 10 mm (total width) × 40 nm (height). Two-point current–voltage scans were recorded at room temperature under ambient atmosphere. The data were recorded using a Keithley (2401) source-meter by sweeping the voltage between −1 and 1 V in hysteresis mode with a 0.1V step. The lateral thin-film conductivity was calculated from Ohm’s law.

## Supporting Information Available

Detailed experimental conditions for the preparation of thin films; additional UV/vis/NIR, LDI-HRMS, and XPS characterizations; and supplementary discussion on the fate of the oxidant during the oCVD reaction and SIMS analysis (PDF)
