# Supplementary material for: Triflate Salts as Alternative Non-Chlorinated Oxidants for the Oxidative Chemical Vapor Deposition and Electronic Engineering of Conjugated Polymers
Source: Macromolecules. 2024 Oct 2;57(20):9627–39. doi: 10.1021/acs.macromol.4c01183 (PMC11500492; doi:10.1021/acs.macromol.4c01183)
Supplement: Supplementary file 1 — ma4c01183_si_001.pdf [file ma4c01183_si_001.pdf]

Supporting Information

Triflate Salts as Alternative Non-Chlorinated Oxidants for the Oxidative Chemical Vapor Deposition and Electronic Engineering of Conjugated Polymers

Drialys Cardenas-Morcoso, Justine Debard, Faezeh Farzin, Nicolas D. Boscher**

Material Research and Technology Department, Luxembourg Institute of Science and Technology, 28, avenue des Hauts-Fourneaux, 4362, Esch-sur-Alzette, Luxembourg

Content

Scheme S1. Representation of the oCVD reactor used for the preparation of conjugated metalloporphyrin polymer thin films. 3

Table S1. Deposition conditions for the chemical vapor deposition of the porphyrin thin films investigated in this work. 4

Figure S1. UV/Vis/NIR absorption spectra of the as-prepared and DCM-rinsed thin films obtained by the oCVD reaction of cobalt(II) 5, 15-diphenyl porphyrin with the different oxidants investigated. The sublimed monomer (prepared in the absence of the oxidant) is included as a reference. 5

Figure S2. a-c) LDI-HRMS spectrum in the dimer and **d-f)** trimer range of the films resulting of the oCVD reaction of CoDPP with the three different oxidants investigated, showing the occurrence of dehydrogenative C–C coupling and side reactions. 6

Figure S3. XPS curve-fitting of the Cl 2p core level for the pCoDPP thin film formed using $FeCl_3$ as the oxidant, showing the contributions of organic and metal chloride environments. 6

Figure S4. Comparison between the LDI-HRMS spectrum (top) and simulated patterns (bottom) in the 1045-1053 region of the thin films prepared using **a)** $Fe(OTf)_3$ and **b)** $Cu(OTf)_2$ as the oxidant. ... 7

Figure S5. Comparison between the LDI-HRMS spectrum of the thin films prepared using $Cu(OTf)_2$ as the oxidant (top) and simulated patterns (bottom) corresponding to homometallic (CoDPP-CoDPP) and (CuDPP-CuDPP), and heterometallic (CoDPP-CuDPP) dimers.. 7

Figure S6. LDI-HRMS spectra of the reference sublimed and oCVD thin films from F-containing cobalt(II) porphyrins, *i.e.* CoD-4-FPP, CoDP-4-CF_3PP and CoDPFPP. 8

Figure S7. Current-voltage plot of oCVD coatings on interdigitated OFET substrates films prepared from **a)** CoDPP and the three different oxidants studied herein, and **b)** CoD-4-FPP, CoDP-4-CF_3PP and CoDPFPP using $FeCl_3$ as oxidant. 9

Supplementary discussion: The fate of the oxidant during the oCVD reaction........................... 10

Figure S8. Secondary electron (SE) map and elemental distribution on **a) sCoDPP** and **b) pCoDPP-($FeCl_3$)**. Field of view is 20×20 μm^2. Image size is (256×256) pixel and acquisition time was 20 ms/pixel.. 12

Figure S9. Secondary electron (SE) map and elemental distribution on **pCoDPP-($Fe(OTf)_3$)** Field of view is 20×20 μm^2. Image size is (256×256) pixel and acquisition time was 20 ms/pixel.................. 15

Figure S10. Secondary electron (SE) map and elemental distribution on **pCoDPP-($Fe(OTf)_3$)** as a function of depth. Field of view is 10×10 μm^2. Image size is (256×256) pixel and acquisition time was 60 ms/pixel.. 16

References.. 17

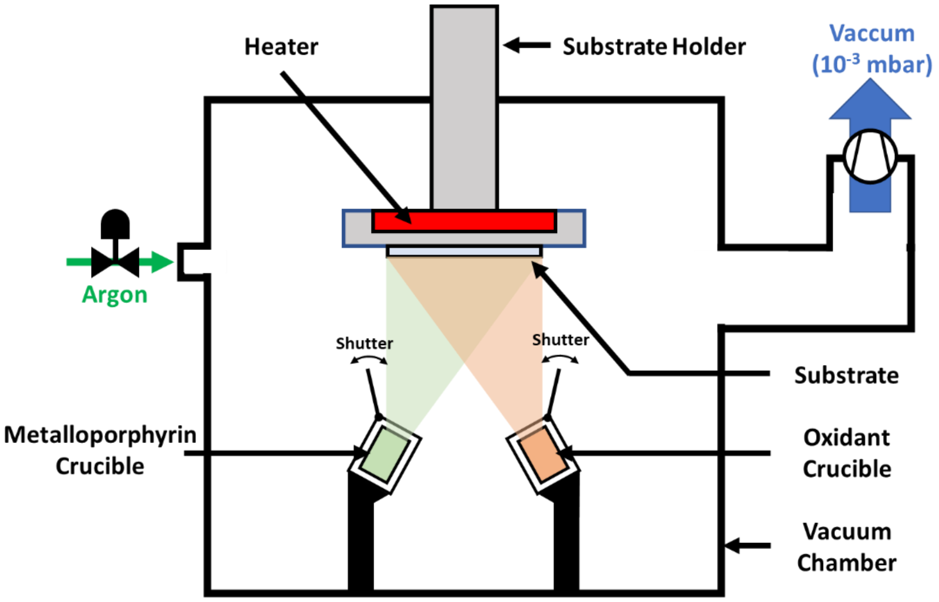

Scheme S1. Representation of the oCVD reactor used for the preparation of conjugated metalloporphyrin polymer thin films.

Table S1. Deposition conditions for the chemical vapor deposition of the porphyrin thin films investigated in this work.

Thin film	T/°C (porphyrin crucible)	Amount of porphyrin loaded /mg	Oxidant	T/°C (oxidant crucible)	Amount of oxidant loaded /mg	Oxidant/porphyrin ratio (consumed)
**sCoDPP**	250	9.9	-	-	-	-
**pCoDPP−($FeCl_3$)**		10.2	**$FeCl_3$**	170	153.2	88.4
**pCoDPP−(Ag(OTf))**		10.4	**Ag(OTf)**	370	207.0	44.4
**pCoDPP−($Fe(OTf)_3$)**		10.3	**$Fe(OTf)_3$**	300	152.6	11.2
**pCoDPP−($Cu(OTf)_2$)**		10.6	**$Cu(OTf)_2$**	320	220.7	10.9
**sCoD-4-CF_3PP**	300	10.1	-	-	-	-
**pCoD-4-CF_3PP−($FeCl_3$)**		9.2	**$FeCl_3$**	170	116.0	49.6
**sCoD-4-FPP**	300	5.0	-	-	-	-
**pCoD-4-FPP−($FeCl_3$)**		7.3	**$FeCl_3$**	170	122.4	57.9
**sCoDPFPP**	275	13.8	-	-	-	-
**pCoDPFPP−($FeCl_3$)**		13.7	**$FeCl_3$**	170	110.2	35.0

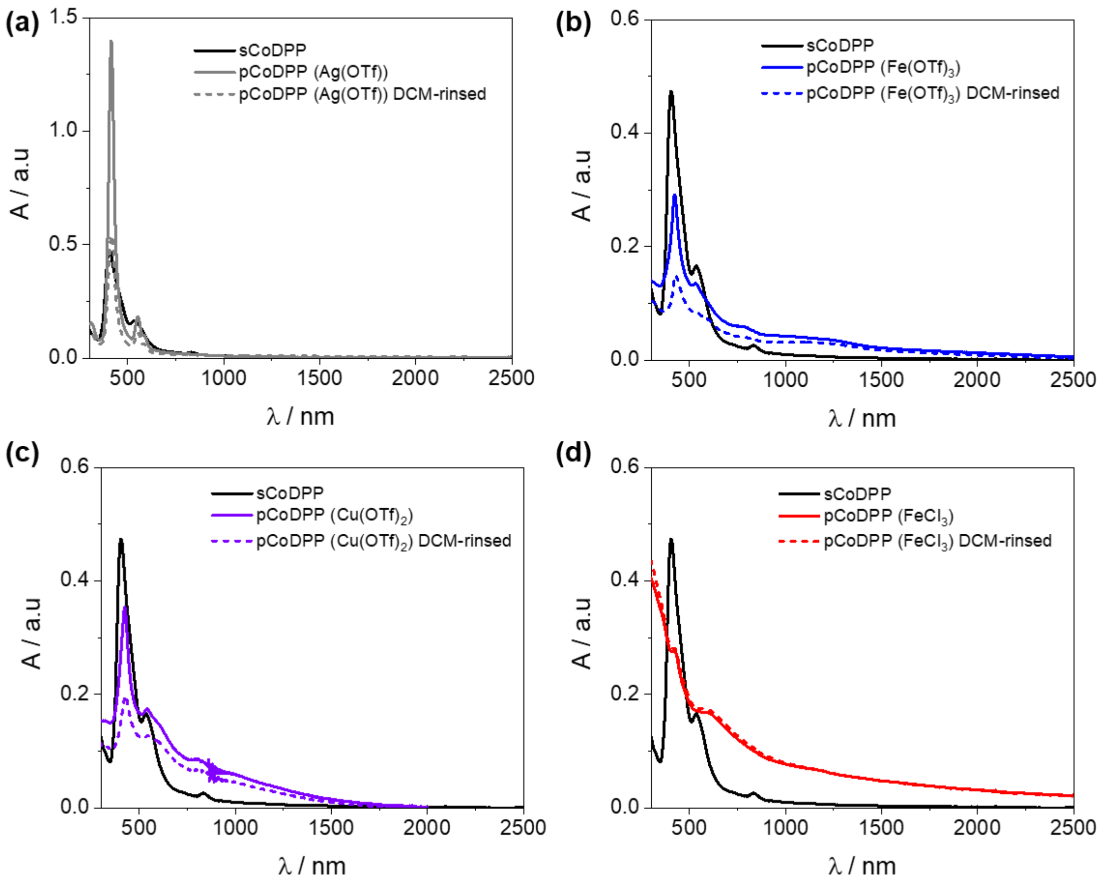

Figure S1. UV/Vis/NIR absorption spectra of the as-prepared and DCM-rinsed thin films obtained by the oCVD reaction of cobalt(II) 5, 15-diphenyl porphyrin with the different oxidants investigated. The sublimed monomer (prepared in the absence of the oxidant) is included as a reference.

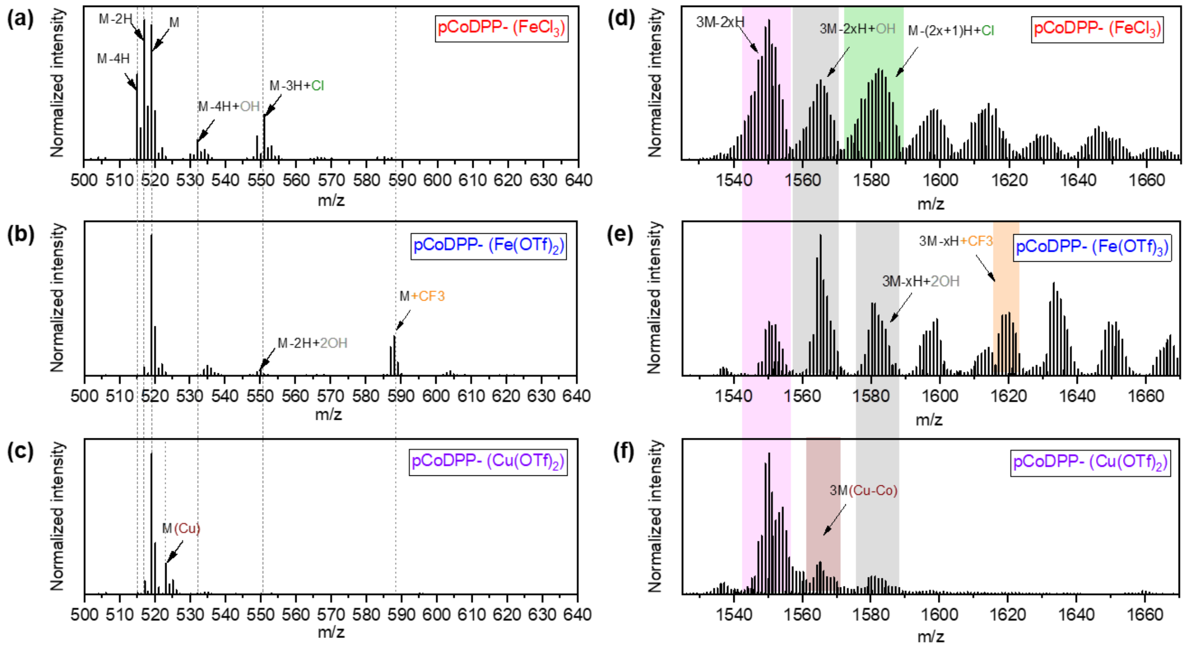

Figure S2. a-c) LDI-HRMS spectrum in the dimer and **d-f)** trimer range of the films resulting of the oCVD reaction of CoDPP with the three different oxidants investigated, showing the occurrence of dehydrogenative C–C coupling and side reactions.

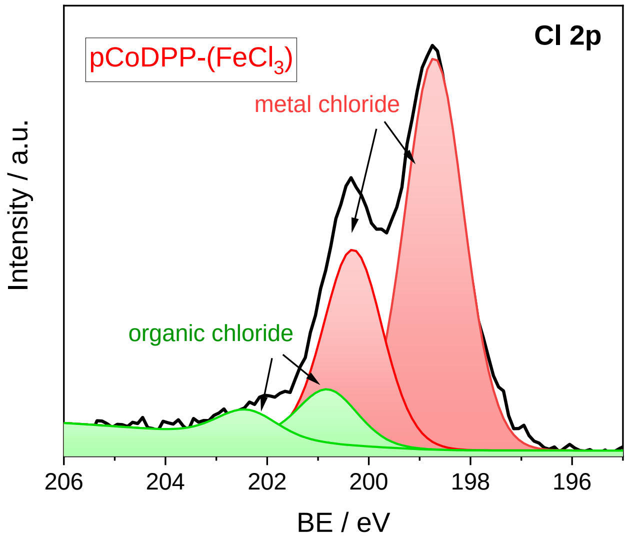

Figure S3. XPS curve-fitting of the Cl 2p core level for the pCoDPP thin film formed using $FeCl_3$ as the oxidant, showing the contributions of organic and metal chloride environments.

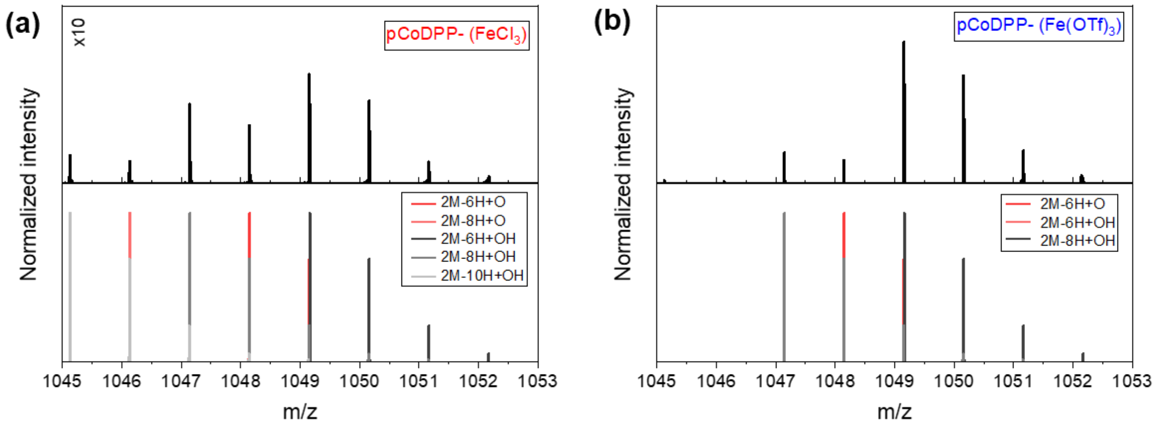

Figure S4. Comparison between the LDI-HRMS spectrum (top) and simulated patterns (bottom) in the 1045-1053 region of the thin films prepared using **a)** $Fe(OTf)_3$ and **b)** $Cu(OTf)_2$ as the oxidant.

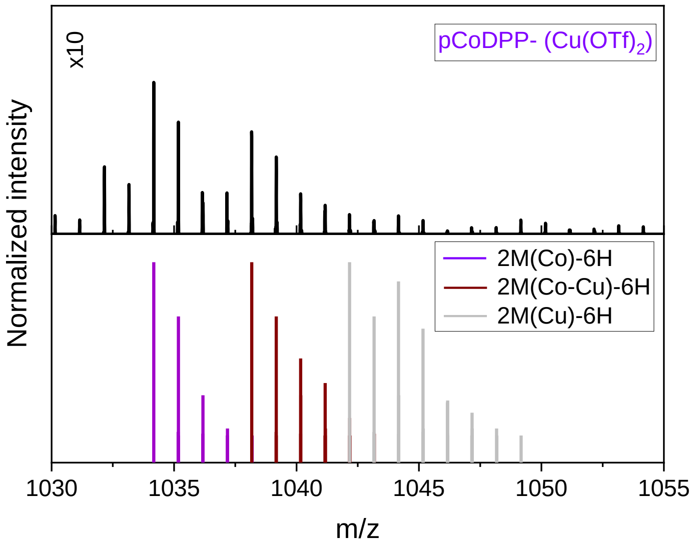

Figure S5. Comparison between the LDI-HRMS spectrum of the thin films prepared using $Cu(OTf)_2$ as the oxidant (top) and simulated patterns (bottom) corresponding to homometallic (CoDPP-CoDPP) and (CuDPP-CuDPP), and heterometallic (CoDPP-CuDPP) dimers.

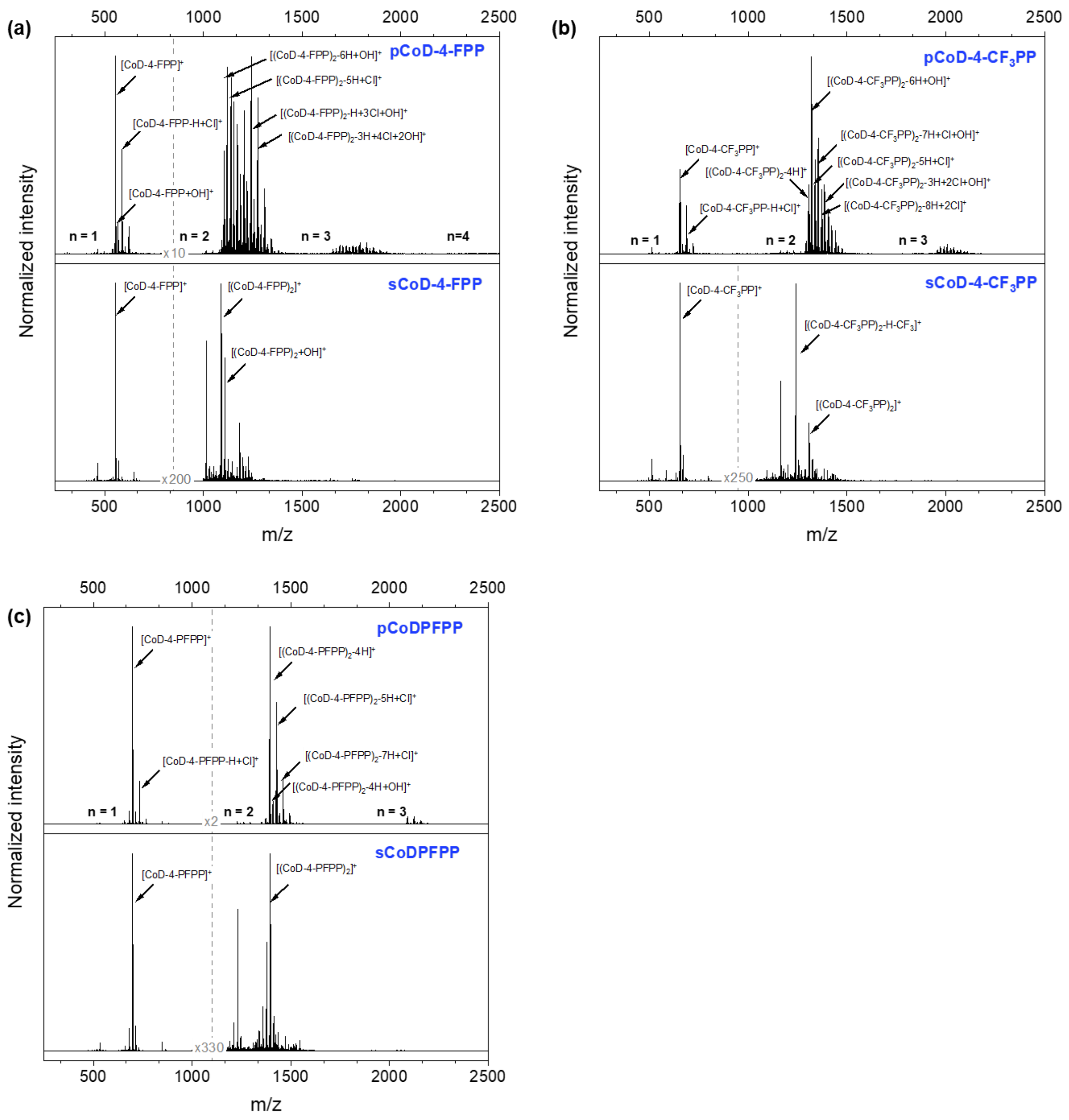

Figure S6. LDI-HRMS spectra of the reference sublimed and oCVD thin films from F-containing cobalt(II) porphyrins, *i.e.* CoD-4-FPP, CoDP-4-CF_3PP and CoDPFPP.

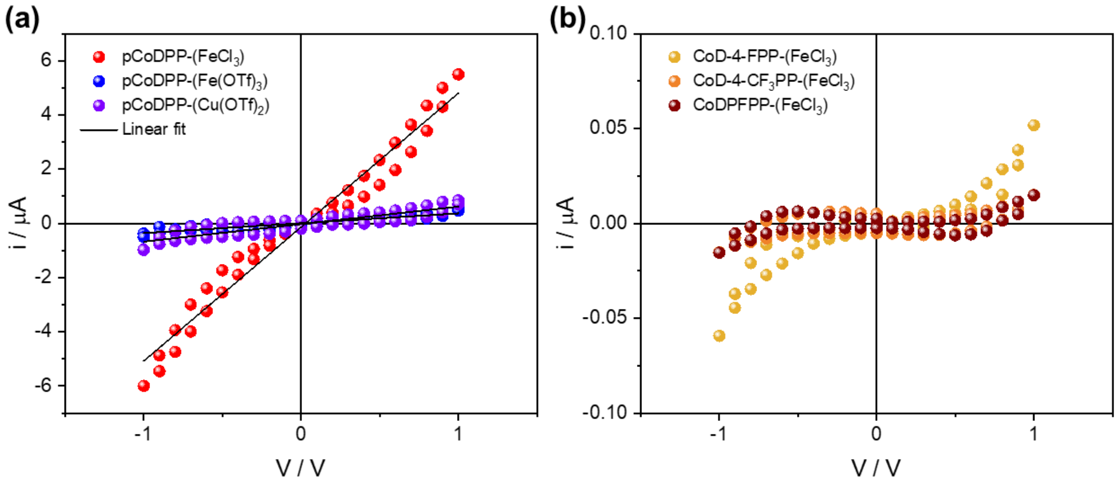

Figure S7. Current-voltage plot of oCVD coatings on interdigitated OFET substrates films prepared from **a)** CoDPP and the three different oxidants studied herein, and **b)** CoD-4-FPP, CoDP-4-CF_3PP and CoDPFPP using $FeCl_3$ as oxidant.

Supplementary discussion: The fate of the oxidant during the oCVD reaction

This section aims to provide further details on the oxidant's fate during and after the oCVD reaction. Not only the role of the oxidant to promote inter- and intramolecular C-C coupling reactions and its related reaction's by-products are considered, but also the possible side reactions leading to inclusion of the oxidant's counter anion to the porphyrin macrocycle and/or the aromatic substituent. Although the following reaction pathways have not been validated experimentally, they propose plausible mechanisms for the incorporation of chloride and trifluoromethyl groups into the conjugated porphyrin polymer, evidenced by the LDI-HRMS analyses. In addition, the possible sublimation and thermal decomposition by-products is discussed.

Iron(III) chloride

During the oCVD reaction, $FeCl_3$ carries out one-electron reduction (see reaction **1** in **Scheme S2**), thereby oxidizing the porphyrin monomer to form a radical cation ($M^{\bullet+}$). The π-radical cation (electron-deficient/electrophile) can readily react with a neutral porphyrin (M) (electron-rich/nucleophile) yielding intermediate adducts (see reaction **2** in **Scheme S2**). A second oxidation and the elimination of two H^+ from the intermediate yields the products of the reaction *i.e., meso-meso, β-meso,* or *β-β* coupled porphyrins.[1] Note that, in the **Scheme S2**, a single *meso-meso* coupling is represented for simplicity. Further oxidation and deprotonation of the dimer yields doubly and triply linked porphyrins. After polymerization reaction, unreacted oxidant and residual Fe^{2+}, Cl^- and H^+ can lead to the by-products $FeCl_{2(s)}$, $HCl_{(s)}$ and $Cl_{2(s)}$.[2-4] While the gas-phase by-products are removed with vacuum pumping, $FeCl_x$ compounds can remain as impurities at the thin film. Concurrently to the dehydrogenative coupling reaction, chloride can react with the porphyrin macrocycle or the aromatic substituent via nucleophilic attack to yield chlorinated species[5] (see reaction **3** in **Scheme S2**).

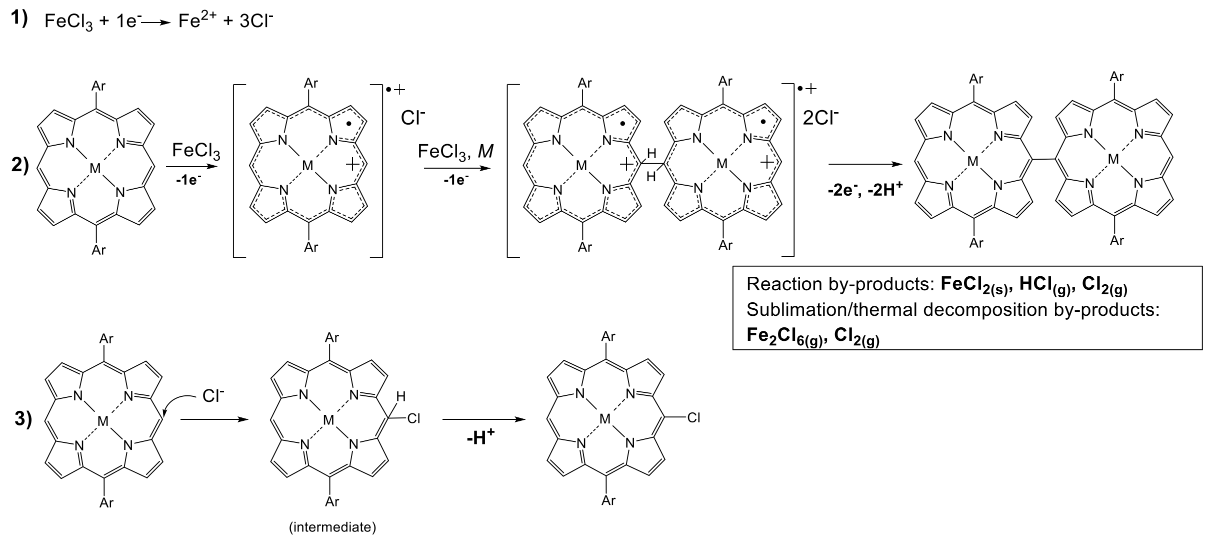

Scheme S2. **1)** One electron reduction of $FeCl_3$, acting as the oxidant agent on the oCVD reaction. **2)** Representation of the intermolecular dehydrogenative C-C coupling reaction of porphyrin units using $FeCl_3$ as the oxidant with the resulting by-products. For simplicity, only meso-meso coupling is represented. **3)** Possible mechanism for chlorination of the porphyrin macrocycle via nucleophilic attack. For simplicity, chlorination is represented with a monomer, however same reaction can take place in porphyrin oligomers during and after C-C coupling, as well as in the aromatic substituents.

It is worth noting that, when sublimating the oxidant salts under vacuum conditions, the reduced pressure generally lowers the sublimation temperature favoring the direct transition of the oxidant specie from solid to gas, while helping to minimize thermal decomposition. However, some decomposition may still occur due to residual heat. In the case of $FeCl_3$, possible sublimation/thermal decomposition by-products in vacuum conditions are $Fe_2Cl_{6(g)}$ and $Cl_{2(g)}$[6].

The presence of oxidant's by-products at the thin film's surface is confirmed through morphological and elemental distribution characterization by Secondary Ion Mass Spectrometry (SIMS) analysis. **Figure 8** shows the secondary electron (SE) and elemental distribution ($^{12}C^{14}N$, ^{35}Cl, $^{56}Fe^{16}O$) images of reference **sCoDPP** and the conjugated polymer **pCoDPP-($FeCl_3$).** The C–N chemical mapping provides evidence of the uniform distribution of the sublimed reference and the conjugated porphyrin polymer across the silicon substrate. Moreover, **pCoDPP-($FeCl_3$)** is characterized by the presence of $FeCl_x$ clusters remaining from the oCVD reaction, as confirmed by the intense Fe and Cl signals of the elemental mapping analysis.

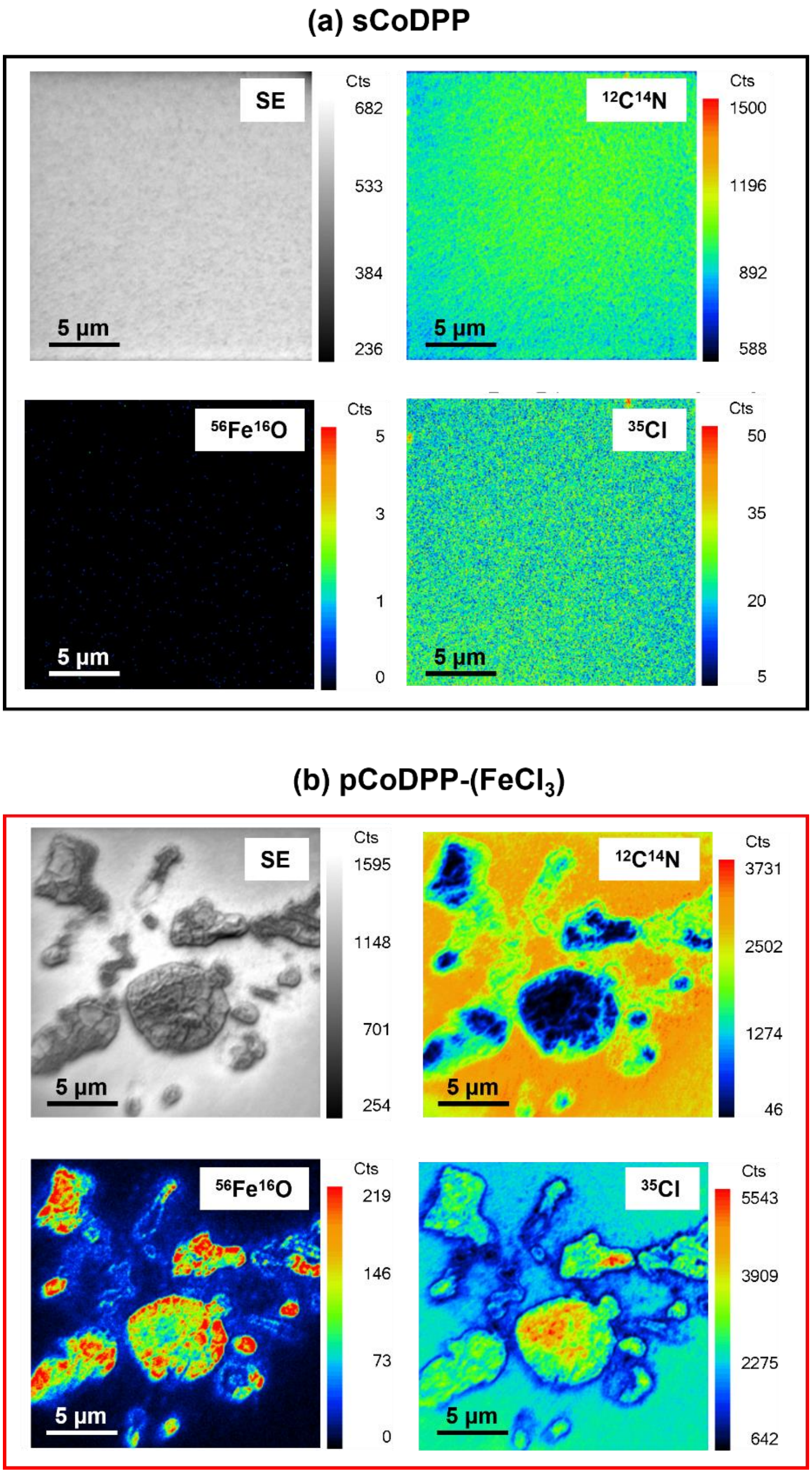

Figure S8. Secondary electron (SE) map and elemental distribution on **a) sCoDPP** and **b) pCoDPP-($FeCl_3$)**. Field of view is 20×20 µm^2. Image size is (256×256) pixel and acquisition time was 20 ms/pixel.

Triflate salts

Similarly to $FeCl_3$, during the oCVD reaction using triflate salts as the oxidant, either $Fe(OTf)_3$ or $Cu(OTf)_2$ carry out one-electron reduction (see reaction **1** in **Scheme S3**), thereby oxidizing the porphyrin monomer to form a radical cation ($M^{\bullet+}$), with the subsequent formation of intermediate adducts and final C-C coupled porphyrins (see reaction **2** in Scheme **S3**). However, the mechanism for the addition of $-CF_3$ groups may differs to the one depicted for chlorination above. Instead, a plausible pathway is the inclusion of $-CF_3$ into the porphyrin macrocycle and/or the aromatic substituents by substitution of a hydrogen atom primarily through a radical mechanism (see reaction **3** in Scheme **S3**).

Indeed, the inclusion of a $-CF_3$ groups into aromatic hydrocarbon compounds is typically facilitated by a radical mechanism.[7, 8] This process involves the generation of a CF_3 radical ($^{\bullet}CF_3$), which can then react with unsaturated compounds with hydrogen loss. This radical mechanism is highly effective due to the reactivity of $^{\bullet}CF_3$, allowing for the efficient incorporation of the $-CF_3$ groups into various organic molecules[9-11], including in this case the porphyrin macrocycle and/or the aromatic substituents. For $^{\bullet}CF_3$ to form during the sublimation of triflate salts, there must be sufficient thermal energy to break the C-S bonds in the triflate anion ($CF_3SO_3^-$).

Herein, $^{\bullet}CF_3$ radicals can be reasonably generated through thermal decomposition of the metal triflate salts during the oCVD reaction. Specifically, vacuum conditions can lower the sublimation temperature of triflate salts, while maintaining sufficient thermal energy to allow the $^{\bullet}CF_3$ formation. Next, $^{\bullet}CF_3$ can undergo an addition reaction with unsaturated bonds of the porphyrin macrocycle/aromatic substituent, forming a radical intermediate. Finally, a hydrogen atom abstraction from the radical intermediate leads to the final CF_3-substituted product (see reaction **3** in Scheme **S3**).

On the other hand, the sublimation/thermal decomposition products of triflate salts can potentially include trifluoromethanesulfonyl fluoride ($CF_3SO_2F_{(g)}$), sulfur oxides ($SO_{2(g)}$ or $SO_{3(g)}$) and metal oxides (*e.g.,* $Fe_2O_{3(s)}$ or $Fe_3O_{4(s)}$).

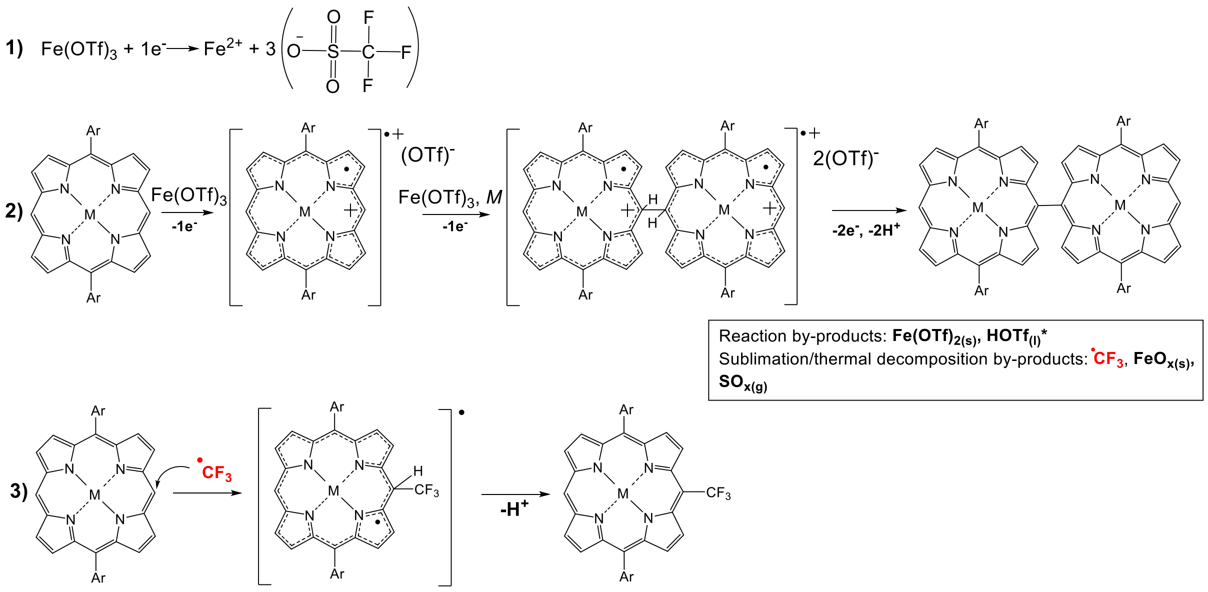

Scheme S3. 1) One electron reduction of $Fe(OTf)_3$, acting as the oxidant agent on the oCVD reaction. **2)** Representation of the intermolecular dehydrogenative C-C coupling reaction of porphyrin units using $Fe(OTf)_3$ as the oxidant with the resulting by-products. For simplicity, only *meso-meso coupling* is represented. **3)** Possible radical mechanism for addition of $-CF_3$ groups to the porphyrin macrocycle. For simplicity, addition of $-CF_3$ is represented with a monomer, however same reaction can take place in porphyrin oligomers during and after C-C coupling, as well as in the aromatic substituents. Although $Fe(OTf)_3$ is used in the representation of C-C coupling and $-CF_3$ addition reaction mechanisms, these are equally valid when using $Cu(OTf)_2$ as the oxidant. (*HOTf is expected to be volatile under oCVD conditions: $T_{(substrate)} = 150°C$, $P = 1 \times 10^{-3}$ mbar)

Figure 9 shows the secondary electron (SE) and elemental distribution ($^{12}C^{14}N$, ^{19}F, $^{56}Fe^{16}O$, ^{32}S, ^{16}O) images of the conjugated polymer **pCoDPP-(Fe(OTf)₃),** obtained by SIMS analysis, evidencing the presence of unreacted oxidant and by-products (likely FeO_x) at the film surface. Nonetheless, the inclusion of F-containing groups in the porphyrin macrocycle/substituents is also evidenced by analysis of the elemental distribution as a function of the sputtering time, *i.e.,* depth, as a constant sputtering rate can be assumed. As showed in **Figure S10**, ^{32}F mapping at lower depth (shorter sputtering time) relates to the presence of F-containing by-products and unreacted oxidant at the thin film surface. However, analysis at higher sputtering time and therefore, deeper inside the sample, reveals a correlation between $^{12}C^{14}N$ and ^{19}F elemental mappings, pointing to the presence of F-containing groups directly related to the porphyrin moieties, consistent with previous LDI-HRMS analysis (**Figure S2**).

pCoDPP-(Fe(OTf)$_3$)

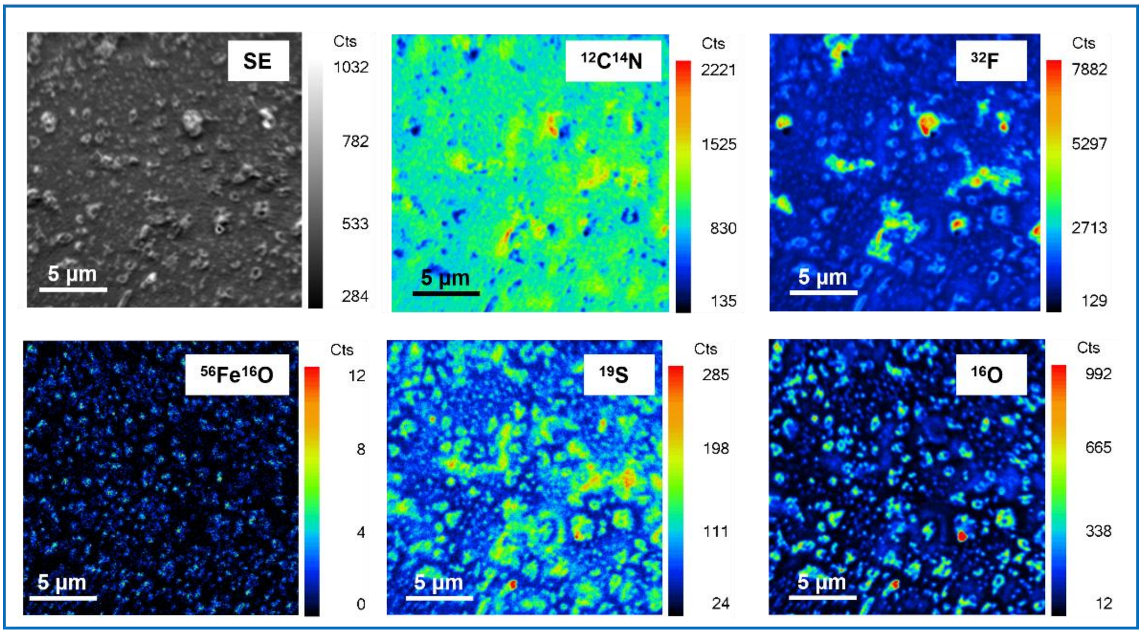

Figure S9. Secondary electron (SE) map and elemental distribution on **pCoDPP-(Fe(OTf)$_3$)** Field of view is 20×20 µm^2. Image size is (256×256) pixel and acquisition time was 20 ms/pixel.

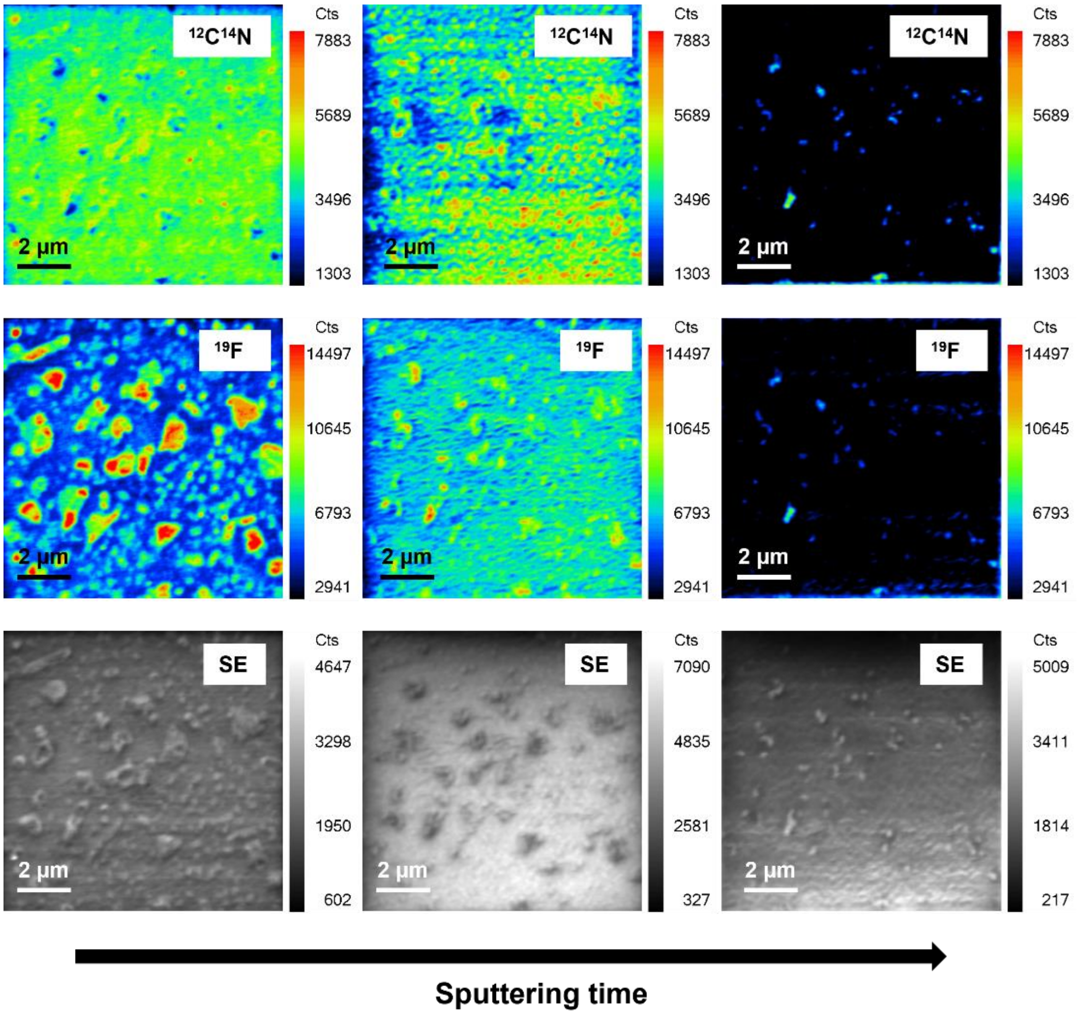

Figure S10. Secondary electron (SE) map and elemental distribution on **pCoDPP-(Fe(OTf)$_3$)** as a function of depth. Field of view is 10×10 μm^2. Image size is (256×256) pixel and acquisition time was 60 ms/pixel.

References

(1) Bengasi, G.; Baba, K.; Back, O.; Frache, G.; Heinze, K.; Boscher, N. D. Reactivity of Nickel(II) Porphyrins in oCVD Processes—Polymerisation, Intramolecular Cyclisation and Chlorination. *Chemistry – A European Journal* **2019**, *25* (35), 8313-8320. DOI: https://doi.org/10.1002/chem.201900793.

(2) Cheng, N.; Zhang, L.; Joon Kim, J.; Andrew, T. L. Vapor phase organic chemistry to deposit conjugated polymer films on arbitrary substrates. *Journal of Materials Chemistry C* **2017**, *5* (23), 5787-5796, 10.1039/C7TC00293A. DOI: 10.1039/C7TC00293A.

(3) Bilger, D.; Homayounfar, S. Z.; Andrew, T. L. A critical review of reactive vapor deposition for conjugated polymer synthesis. *Journal of Materials Chemistry C* **2019**, *7* (24), 7159-7174, 10.1039/C9TC01388A. DOI: 10.1039/C9TC01388A.

(4) Atanasov, S. E.; Losego, M. D.; Gong, B.; Sachet, E.; Maria, J.-P.; Williams, P. S.; Parsons, G. N. Highly Conductive and Conformal Poly(3,4-ethylenedioxythiophene) (PEDOT) Thin Films via Oxidative Molecular Layer Deposition. *Chemistry of Materials* **2014**, *26* (11), 3471-3478. DOI: 10.1021/cm500825b.

(5) F. Mironov, A.; D. Rumyantseva, V.; N. Ponamoreva, O. A porphyrin chlorination reaction. *Mendeleev Communications* **1998**, *8* (5), 187-188, 10.1070/MC1998v008n05ABEH000951. DOI: 10.1070/MC1998v008n05ABEH000951.

(6) Blairs, S. Sublimation study of anhydrous ferric chloride. *The Journal of Chemical Thermodynamics* **2006**, *38* (11), 1484-1488. DOI: https://doi.org/10.1016/j.jct.2005.12.012.

(7) Ji, Y.; Brueckl, T.; Baxter, R. D.; Fujiwara, Y.; Seiple, I. B.; Su, S.; Blackmond, D. G.; Baran, P. S. Innate C-H trifluoromethylation of heterocycles. *Proceedings of the National Academy of Sciences* **2011**, *108* (35), 14411-14415. DOI: doi:10.1073/pnas.1109059108.

(8) Stefani, A. P.; Szwarc, M. Addition of CF3 Radicals to Aromatic Hydrocarbons. The Relative Selectivity of CF3. *Journal of the American Chemical Society* **1962**, *84* (19), 3661-3666. DOI: 10.1021/ja00878a012.

(9) Ma, J.-A.; Cahard, D. Strategies for nucleophilic, electrophilic, and radical trifluoromethylations. *Journal of Fluorine Chemistry* **2007**, *128* (9), 975-996. DOI: https://doi.org/10.1016/j.jfluchem.2007.04.026.

(10) Studer, A. A "Renaissance" in Radical Trifluoromethylation. *Angewandte Chemie International Edition* **2012**, *51* (36), 8950-8958. DOI: https://doi.org/10.1002/anie.201202624.

(11) Song, H. Research progress on trifluoromethyl-based radical reaction process. In *IOP Conference Series: Earth and Environmental Science*, 2017; IOP Publishing: Vol. 100, p 012061.
